# Non-invasive Intrauterine Administration of Botulinum Toxin A Enhances Endometrial Angiogenesis and Improves the Rates of Embryo Implantation

**DOI:** 10.1007/s43032-021-00496-4

**Published:** 2021-03-01

**Authors:** Hwa Seon Koo, Min-Ji Yoon, Seon-Hwa Hong, Jungho Ahn, Hwijae Cha, Danbi Lee, Chan Woo Park, Youn-Jung Kang

**Affiliations:** 1 CHA Fertility Center Bundang, 59, Yatap-ro, Bundang-gu, Seongnam-si, Gyeonggi-do South Korea; 2 Department of Biomedical Science, School of Life Science, CHA University, 335 Pangyo, Bundang-gu, Seongnam-si, Gyeonggi-do South Korea; 3 Department of Biochemistry, Research Institute for Basic Medical Science, School of Medicine, CHA University, 335 Pangyo, Bundang-gu, Seongnam-si, Gyeonggi-do South Korea; 4 CHA Fertility Center Gangnam, 566, Nonhyeon-ro, Gangnam-gu, Seoul, South Korea

**Keywords:** Endometrial receptivity, Embryo implantation, Botulinum toxin A, Non-invasive treatment, Angiogenesis

## Abstract

Endometrial angiogenesis plays crucial roles in determining the endometrial receptivity. Defects in endometrial receptivity often cause repeated implantation failure, which is one of the major unmet needs for infertility and contributes a major barrier to the assisted reproductive technology. Despite the numerous extensive research work, there are currently no effective evidence-based treatments to prevent or cure this condition. As a non-invasive treatment strategy, botulinum toxin A (BoTA) was administered into one side of mouse uterine horns, and saline was infused into the other side of horns for the control. Impact of BoTA was assessed in the endometrium at 3 or 8 days after infusion. We demonstrated that BoTA administration enhances the capacity of endothelial cell tube formation and sprouting. The intrauterine BoTA administration significantly induced endometrial angiogenesis displaying increased numbers of vessel formation and expression levels of related marker genes. Moreover, BoTA intrauterine application promoted the endometrial receptivity, and the rates of embryo implantation were improved with BoTA treatment with no morphologically retarded embryos. Intrauterine BoTA treatment has a beneficial effect on vascular reconstruction of functional endometrium prior to embryo implantation by increasing endometrial blood flow near the uterine cavity suggesting BoTA treatment as a potential therapeutic strategy for patients who are suffering from repeated implantation failure with the problems with endometrial receptivity.

**Supplementary Information:**

The online version contains supplementary material available at 10.1007/s43032-021-00496-4.

## Introduction

Angiogenesis is the process of new capillary formation from existing vascular structure by elongation or sprouting of endothelial cells, and is tightly regulated since most organs in the adult usually do not undergo physiological angiogenesis unless they are not injured by trauma or infection [1–3], while the female reproductive tract requires continuous and cyclic angiogenesis for the growth of primordial follicles during the ovulation, receptive endometrium for the embryo implantation, and placental and embryonic development for successful pregnancy [4–6]. In particular, endometrial angiogenesis is a fundamental process for the menstrual cycle, endometrial receptivity, and embryo implantation [7]. Prior to embryo implantation, the endometrium undergoes dynamic changes of shedding and repair induced by ovarian steroid hormones to produce a period of uterine receptivity referred to as the “window of implantation” [8–10]. During the menstrual cycle, endometrial vasculature experiences continuous growth and regression, and coordinated vascular development induced by endometrial angiogenesis is essentially required for successful embryo implantation [11]. Direct or indirect association between implantation success and the endometrial angiogenesis facilitated by the conformational changes of end arterioles arising from arcuate arteries still need to be elucidated. Recent studies showing the increased expression levels of angiogenesis-related factor, VEGF, and its receptor in the late secretory and early pregnancy strongly suggest that the endometrial angiogenesis plays crucial roles in endometrial response to the implanting blastocyst, likely a key factor of endometrial vasculature in determining the endometrial receptivity [3, 12]. Even though there are several trials to improve endometrial environment via inducing angiogenic effect with many kinds of substances including granulocyte colony-stimulating factor (G-CSF), mesenchymal stem cells (MSC), and platelet-rich plasma (PRP) [13–15], the efficacy of those substances is still controversial due to the lack of evidences [16, 17].

Botulinum toxin A (BoTA) is widely used in the plastic, reconstructive, and aesthetic surgery fields [18, 19]. In addition to these usages, BoTA is commonly used in medical applications to treat chronic myofascial pain, headache, urinary incontinence, and hyperhidrosis [20–23]. Lately, there have been reports demonstrating the effect of BoTA on flap survival, which was suggested to occur by increased vasodilation and angiogenesis via elevated hypoxia-inducible factor (HIF)1α and vascular endothelial growth factor (VEGF) [24, 25]. Moreover, BoTA is reported to enhance re-epithelialization of human keratinocytes and angiogenesis of endothelial cells [26]. However, there was no report that evaluates the therapeutic effect of BoTA in the endometrium, especially endometrial angiogenesis, regarding the reproductive functionality. This led us to explore the impact of BoTA on endometrial angiogenesis and further investigation into the capacity of BoTA for the rates of embryo implantation for successful pregnancy via elevation of endometrial receptivity.

## Materials and Methods

### Cell Culture and Botulinum Toxin A Treatment

Human umbilical vein cells (HUVEC, ATCC) and human uterine microvascular endothelial cells (HUtMEC, PromoCell) were maintained in EGM^TM^-2 endometrial cell growth medium 2 (Lonza) and endothelial cell growth medium MV (PromoCell), respectively. Ishikawa (ATCC) and CRL-4003 cells (kindly gifted from the laboratory of Dr. Haeng Seok Song) were maintained in DMEM/F12 media (Gibco, Grand Island, NY, USA) supplemented with 10% fetal bovine serum (Gibco, Grand Island, NY, USA) and 1% penicillin-streptomycin (Gibco, Grand Island, NY, USA) as previously described [27]. All experiments were conducted using cells of the passages between 1 and 10. For the analyses for the effects of BoTA, cells were treated with commercially available BoTA (Botulax, Hugel, Seoul, Korea) for 24h, 48h, or 72h at concentration of 0.5, 2.0, 10, or 20IU. Botulax is the type for Clostridium botulinum toxin A derived from the strain of Clostridium Botulinum CBFC26, and for the utilization of BoTA from Botulax (Hugel) to perform the all analyses, the powder form of BoTA was dissolved and diluted in sterile 0.9% saline solution.

### Tube Formation Assays

Assessment of the rates of endothelial tube formation in the presence or absence of BoTA (0, 0.5 IU, and 2.0 IU) was performed in a 96-well plate. 2×10^4^ HUVECs or HUtMECs were seeded onto growth factor reduced Matrigel matrix (#354230, Corning Inc., Corning, NY, USA) pre-coated wells in 100ul of EGM^TM^-2 endometrial cell growth medium 2 and endothelial cell growth medium MV, respectively. Following incubation at 37°C overnight, each well was analyzed directly under a microscope. The images (×10 magnification) were subsequently analyzed using ImageJ.

### Sprouting Assays

**Fig. 1:**
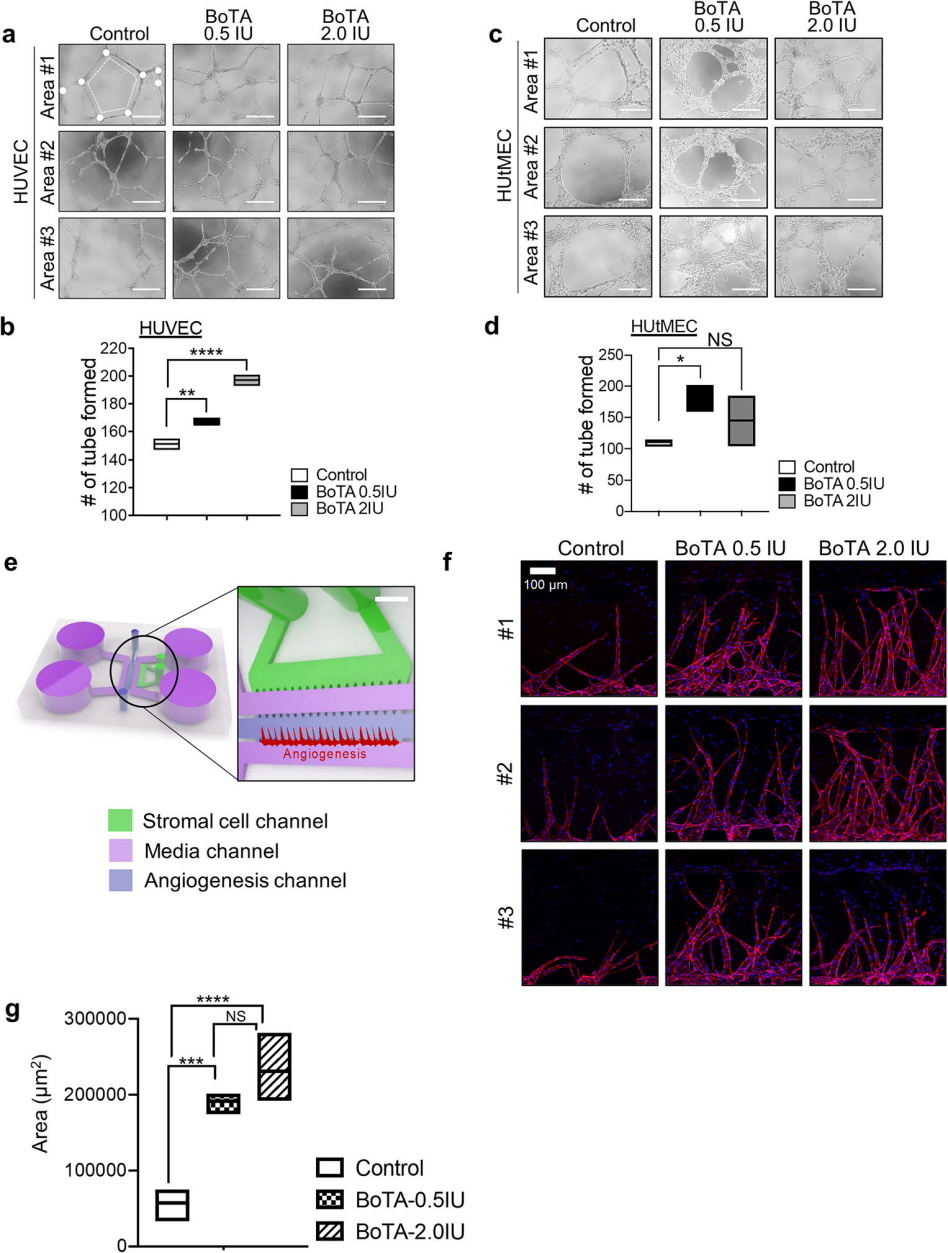
The impact of BoTA treatment on the capacity of tube formation and sprouting of endothelial cells. Representative three images showing tube formation of HUVECs (**a–b**) and HUtMECs (**c–d**) in the condition of BoTA (0, 0.5, 2.0IU) treatment for 48h. Scale bar: 200um. The total number of tube loops was quantified in graphs shown in (**b)** and (**d**). (**e**) A schematic diagram of a micro-engineered vascular system that was used for the analyses of angiogenic effect of BoTA. CRL4003 stromal cells were mixed in fibrin matrix and then plated in the stromal cell channel. HUVECs attached on the wall of fibrin gel were patterned in the central channel. Scale bar: 800 μm. (**f**) Representative confocal microscopic images of angiogenic sprouting of HUVECs in response to BoTA. Scale bar: 100 μm. The total surface area of HUVEC sprouting was quantified in a graph shown in (**g**). Data shown in (**b**), (**d**), and (**g**) are from 3 independent experiments and analyzed using the ordinary one-way ANOVA analysis with Dunnett’s multiple comparison test including *P*-values (*<0.05, **<0.01, ***<0.001, ****<0.0001, *NS* not significant)

Microfluidic device with the center channel of 800μm of width was used for HUVEC sprouting assays that were previously described [28]. The microfluidic chips were fabricated by polydimethylsiloxane (PDMS, Sylgard 184, Dow Corning) mold embedded with channel structures that was patterned by standard photolithography photoresist SU-8 (MicroChem). Demolded PDMS was punched out by using a biopsy punch (6mm) and sharpened syringe needle (0.5mm) to make reservoirs for the medium and hydrogel injection ports. A PDMS device and a glass coverslip were treated with oxygen plasma for 1 min before bringing them into contact. The devices were incubated in an 80°C dry oven for at least 48h to restore hydrophobicity of PDMS. The devices were sterilized by UV irradiation before use. CRL4003 (8×10^6^/ml) cells were mixed with fibrinogen solution (2.5 mg/ml fibrinogen with 0.15 U/ml aprotinin). Thrombin (0.5 U/ml) was added to cell mixture and immediately introduced into the channel for stromal cell. Fibrinogen solution (2.5 mg/ml of fibrinogen with 0.15 U/ml of aprotinin) mixed with thrombin (0.5 U/ml) was applied to the vessel channel. After 3min, the upper reservoirs of each device were filled with EGM-2 culture medium and aspirated gently from the lower reservoirs to wet the hydrophobic media channel. Subsequently, HUVECs (5×10^6^/ml) were plated into the media channel. The device was then tilted 90° in an incubator for 40 min to attach the cell mixture to the gel-media interface. The device with multilayer of cells was incubated for 7 days until fully lumenized microvessels had formed. A schematic illustration of cell loading and generation of perfused microvessel on the device is shown in Fig. 1. To quantify the area for angiogenic sprouting of HUVECs, cells were immunostained against CD31 (abcam; ab28364, 1:100) and detected its expressed area using confocal microscope. Z-projections of the 3D stacks of microvascular networks were obtained each day with IMARIS (Bitplane) and further analyzed with ImageJ (National Institutes of Health, Bethesda, MD) to obtain the binary images. The proportion of fluorescent pixels within the region of interests of each image was calculated.

### Quantitative RT-PCR-Based Analysis of mRNA Expression

SYBR Green (Roche, Basel, Switzerland) assays were used to quantitate angiogenesis- and embryo implantation-related genes in BoTA-treated and control samples. Total RNA extracted using TRIzol reagent (Ambion, Life Technologies Corporation, CA, USA) at 1ug was converted to cDNA using M-MLV reverse transcriptase (Promega, Madison, WI, USA), dNTP (Invitrogen, Carlsbad, CA, USA), and oligo dT primer (Labopass, Seoul, Korea). The efficiency of the primer sequences was tested by creating an external standard curve. Synthesized cDNA was sequentially diluted with the dilution factor 1:10 four times (1/1, 1/10, 1/100, and 1/1000). Amplifications were run where each primer pair was loaded into wells containing different cDNA concentrations in a CFX Connect^TM^ Real-Time PCR Detection System (BioRad, Hercules, CA, USA). A standard curve was generated for each primer by plotting the log value of the starting quantity of template against the cycle threshold value obtained during the amplification of each dilution displaying *R*^2^ value greater than 0.98 and an amplification efficiency around 100%. With 1/10 volume of cDNA, gene expression was quantitatively analyzed. A DNA melting-curve was used to confirm the presence of a single PCR product in each assay. Real-time PCR results for angiogenesis- and embryo implantation-related genes, which were measured by C_T_ values, were normalized to C_T_ values of β-actin and evaluated as a percentage of expression levels measured in cells of control group (the ratio for interesting gene/β-actin is quantified by 2^CT(interesting gene)-CT(β-actin)^). All values were analyzed using the ordinary one-way ANOVA analysis with Dunnett’s multiple comparison tests. Primer sequence pairs used for these analyses are shown in Supplementary Table 1.

### Immunofluorescence and Microscopy

Immunofluorescence staining was performed as previously described [29]. Cells were fixed with 4% of paraformaldehyde and were specified and permeabilized for 15 min using 0.05%Triton X-100/PBS. Subsequently, cells were blocked using 5% w/v bovine serum albumin (BSA) in PBS for 1h at room temperature. Localization studies were performed using primary antibody to HIF1-α (Cell signaling technology; #3434, 1:50), integrin αvβ3 (MILLIPORE; MAB1976, 1:100), LIF (NovusBiologicals; NBP1-85717, 1:100), CD31 (abcam; ab28364, 1:100), and CD34 (abcam; ab8536, 1:100) and further incubated with anti-rabbit IgG fluorescence (Invitrogen) or anti-mouse IgG fluorescence (Invitrogen) as appropriate, all at 1:400 for 1h. Cover glasses were mounted in Vectashield mountant with 4′,6-diamidino-2-phenylindole (DAPI; Vector Laboratories) as nuclear stain. For the negative control, cells were incubated under similar conditions with isotype-matched mouse or rabbit immunoglobulin in the place of the primary antibodies. Images were captured using oil immersion 63x objectives Zeiss 510 microscopy (Carl Zeiss MicroImaging, Röttingen, Germany) and processed using Zen software (ZEISS).

### Immunohistochemistry

Histology and immunohistochemistry were performed as previously described [27]. Tissues were fixed with 4% of paraformaldehyde and embedded in paraffin. Paraffin-embedded tissue sections (5μm) were deparaffinized in Histoclear (National diagnostics; HS-202) and dehydrated with gradient absolute ethanol (80–100%) for 3 min each. Antigen retrieval was performed in sodium citrate buffer (pH 6.0) for 1h at 95°C. Endogenous peroxidase activity was blocked using 3% H_2_O_2_/PBS for 10 min at room temperature and washed with PBS. Subsequently, non-specific binding sites were blocked with 1% BSA/PBS for 1h. Sections were incubated overnight at 4°C with primary antibody to integrin β3 (Cell Signaling; #13166, 1:200) and OPN (Enzo; ADI-905-629, 1:100). After three washes with PBS, sections were incubated with biotinylated goat anti-rabbit secondary antibodies (Vector laboratories; ZG0122 1:200) for 30 min at room temperature. The signal was detected using Avidin-Biotin HRP (Vector Laboratories; DK-6100, Vectastain Elite ABC Kit) with DAB substrate solution (Thermo; VE296420, DAB substrate kit). Sections were counterstained with hematoxylin (BioGNOST; HEMH-46/20), dehydrated in a graded ethanol for 3 min each, cleared in Histoclear, and mounted with Permount medium (Fisher chemical; 196934). For the negative control, cells were incubated under similar conditions with isotype-matched mouse or rabbit immunoglobulin in the place of the primary antibodies. Images were captured using Olympus CKX53 microscopy (Olympus Life Science) and processed using eXcope.

### Animal Uses and BoTA Infusion

All experiments were conducted under a Home Office license and the Animal Act, 1986, and had local ethical approval for care and use of laboratory animals. C57BL/6 strain mice were maintained by strict accordance with the policies of the CHA University Institutional Animal Care and Use Committee (IACUC, approval number 190126). All mice were housed under standard environmental conditions of 12h light:12h dark at a controlled room temperature (20–22°C and 40–60% humidity) and fed *ad libitum*. Five-week-old female and 7-week-old male mice provided by Orientbio (Gapyeong, Gyeonggi, South Korea) were used for the analyses and evaluation of impact of BoTA. The female mice were anesthetized via intraperitoneal injection of tribromoethanol (Avertin). A vertical incision was made to gently expose the uterus through the abdominal wall. BoTA was prepared in 30ul of saline and softly instilled into one side of mouse uterine cavities through the uterotubal junction using an insulin syringe equipped with a 31-gauge needle, and saline was infused into the other side of horns for the control. The overall operation time of a mouse was no more than 15 min, and the unilateral uterine horn administration time was about 5 s. After 8 days of BoTA infusion, female mice were weakly stimulated for the ovulation with 2.5IU of pregnant mare serum gonadotropin (PMSG, Daesung Microbiological, Korea), and ovulation was synchronized by 1.25IU of human chorionic gonadotropin (hCG, Sigma, USA) 46–48h later, both by intraperitoneal injection. Females were placed singly with the same strain male mice overnight. The presence of a vaginal plug the following morning (day 1 of pregnancy) was used as an indicator of successful mating. Both sides of uterine horns were obtained 16 days after mating for further analyses of pregnancy outcomes. Implantation sites and embryos were isolated, and the total numbers were counted to analyze the rates of embryo implantation. Isolated embryos were weighed individually, and their individual sizes and growth morphology were examined.

### Mouse Embryo Collection and Co-culture

For the embryo collection, female mice (6–8 weeks) were superovulated with 10IU of PMSG, and ovulation was synchronized by 5IU hCG 46–48h later, both by intraperitoneal injection. Females were placed singly with males of the same strain overnight. The morning of the presence of a vaginal plug was designated day 1 of pregnancy. Pregnant mice were killed on day 1. One-cell embryos were obtained from the oviduct using a 30G dissecting needle of 1ml syringe to tear open the ampulla of the oviduct and release. Cumulus cell around of the one-cell embryo were washed with 0.1% hyaluronidase drop at 37°C for 5–10 min and dissociated by gentle pipetting to glass pipettes. Collected embryos were washed with M2 media (Sigma, USA) supplemented with 4 mg/ml BSA washed in KSOM (Millipore, USA) and cultured in a 20ul drop of KSOM covered with mineral oil at 5% CO_2_, 37°C until the blastocyst stage. Only expanded blastocysts with clearly observable inner cell mass and trophectoderm on day 5 were included in the study. Day 5 mouse embryos were transferred onto saline- or BoTA-treated Ishikawa cells in independent wells of 24-well plate. Multiple observations of stability for embryo attachment were performed to identify the distinct stages of attachment from 12 to 48h of co-culture as previously reported [27, 29]. Standardized plate movement protocol was applied to assess the stability of embryo attachment. The plate was tapped three times laterally and orthogonally to detect unattached embryos. Five stages of attachment were defined and used as a measurement scale as previously described [29, 30].

### Library Preparation and Sequencing for RNA-Seq Analysis

For control and test RNAs, the construction of library was performed using QuantSeq 3’ mRNA-Seq Library Prep Kit (Lexogen, Inc., Austria) according to the manufacturer’s instructions. In brief, each 500ng total RNA was prepared, and an oligo-dT primer containing an Illumina-compatible sequence at its 5’ end was hybridized to the RNA, and reverse transcription was performed. After degradation of the RNA template, second strand synthesis was initiated by a random primer containing an Illumina-compatible linker sequence at its 5’ end. The double-stranded library was purified by using magnetic beads to remove all reaction components. The library was amplified to add the complete adapter sequences required for cluster generation. The finished library is purified from PCR components. High-throughput sequencing was performed as single-end 75 sequencing using NextSeq 500 (Illumina, Inc., USA). The raw and normalized data have been deposited in the Gene Expression Omnibus (GEO) database (accession number: GSE146934, Supplementary Table 2).

### Data Analysis

QuantSeq 3’ mRNA-Seq reads were aligned using Bowtie2 (Langmead and Salzberg, 2012). Bowtie2 indices were either generated from genome assembly sequence or the representative transcript sequences for aligning to the genome and transcriptome. The alignment file was used for assembling transcripts, estimating their abundances and detecting differential expression of genes. Differentially expressed genes were determined based on counts from unique and multiple alignments using coverage in Bedtools (Quinlan AR, 2010). The RC (read count) data were processed based on quantile normalization method using EdgeR within R (R development Core Team, 2016) using Bioconductor (Gentleman *et al*., 2004). Gene classification for gene ontology (GO) and pathway analysis was performed by DAVID (http://david.abcc.ncifcrf.gov/) and Medline databases (http://www.ncbi.nlm.nih.gov/). Classified genes and their fold enrichment values were visualized into dotplots by using R and automatically categorized by ClueGO [31, 32]. The significance cutoffs were set for fold change (≥2.0), *P*-value (<0.05), and FDR (<0.05).

### Cell Proliferation and Cytotoxicity Assay

Cell proliferation assay of Ishikawa cells was performed by cell proliferation assay kit (BIOMAX, Seoul, Korea) according to the manufacturer’s instructions. 1×10^4^ cells were seeded and maintained in the presence or absence of BoTA at indicated concentrations for 24h, 48h, or 72h. Absorbance was measured using microplate reader (Thermo Scientific, Rockford, IL, USA) at 450nm wavelength followed by adding 10ul QuantiMAX™ mixture (BIOMAX, Seoul, Korea). Cytotoxicity of Ishikawa cells depending on BoTA treatment was measured using EZ-LDH^TM^ cytotoxicity assay kit (BIOMAX, Seoul, Korea) according to the manufacturer’s instructions. 5×10^3^ Ishikawa cells were treated with BoTA at indicated concentrations for 24h, 48h, or 72h, and absorbance was measured by adding 10ul of LDH reaction mixture. Reaction of LDH mixture was performed on a shaker for 90min, and then the number of survived cells was counted.

### Wound Healing Assay

Ishikawa cells were seeded in 6-well plate and cultured to 100% confluence. After 24h of starvation, a linear scratch was generated using a sterile 1ml pipette tip. Cells were treated with BoTA at indicated concentrations for 48h. Images of wound closure were taken using inverted microscope (Olympus Corp., Tokyo, Japan), and the gap distances of wound closure were analyzed using ImageJ.

### Statistical Analysis

Comparison groups were analyzed with unpaired Student *t*-test for parametric distributions. For multiple comparisons, the ordinary one-way ANOVA analysis with Dunnett’s multiple comparison test was used. For all cases, a *P*-value that was <0.05 was considered statistically significant (*P*<0.05(*), *P*<0.01(**), *P*<0.001(***), and *P*<0.0001(****)).

## Results

### BoTA-Induced Enhancement of Angiogenesis in Endothelial Cells *In Vitro*

To investigate the effect of BoTA treatment on the tube formation ability of human endothelial cells, we plated HUVECs onto Matrigel-coated plate, and 0.5IU or 2.0IU of BoTA was applied. The tube formation as measured by the total number of tube loops (indicated with dash line in the image) and branching points (indicated with white dots in the image) was significantly higher in BoTA-treated HUVECs compared to saline-treated controls (Fig. 1a–b). More specifically, the assessment of tube formation of human uterine endothelial cells (HUtMEC) revealed that BoTA induced the angiogenic effect exhibiting dramatically higher numbers of tube formation in BoTA (0.5IU)-treated groups compared to control (Fig. 1c–d). Interestingly, unlike HUVECs, HUtMECs showed no further significant effect in tube formation at higher dose (2.0IU) of BoTA treatment. Furthermore, we implemented a micro-engineered 3-dimensional angiogenesis system to explore the effect of BoTA on the ability of vessel formation and sprouting using HUVECs and endometrial stromal CRL4003 cells. The device consists of 5 primary channels; 2 fluidic microchannels separate 3 hydrogel-laden microchannels from each other to facilitate the supply of fresh media through the device (Fig. 1e). This co-culture system enables paracrine interaction between HUVECs and endometrial stromal layer, mediating the angiogenic morphogenesis. The micro-engineered angiogenesis model was exposed to BoTA at 0.5IU or 2.0IU. These analyses revealed that BoTA treatment induced an increasing effect on angiogenesis displaying promoted angiogenic sprouting of HUVECs in BOTA-treated group compared to control (0.5IU; *P*=0.0003, 2.0IU; *P*<0.0001) (Fig. 1f), which is consistent with the data from tube formation assay. This increasing angiogenic effect was further quantified through the analysis of the sprout area (Fig. 1g).

### BoTA-Induced Elevation of Endometrial Receptivity Markers in Endometrial Cells *In Vitro*

**Fig. 2:**
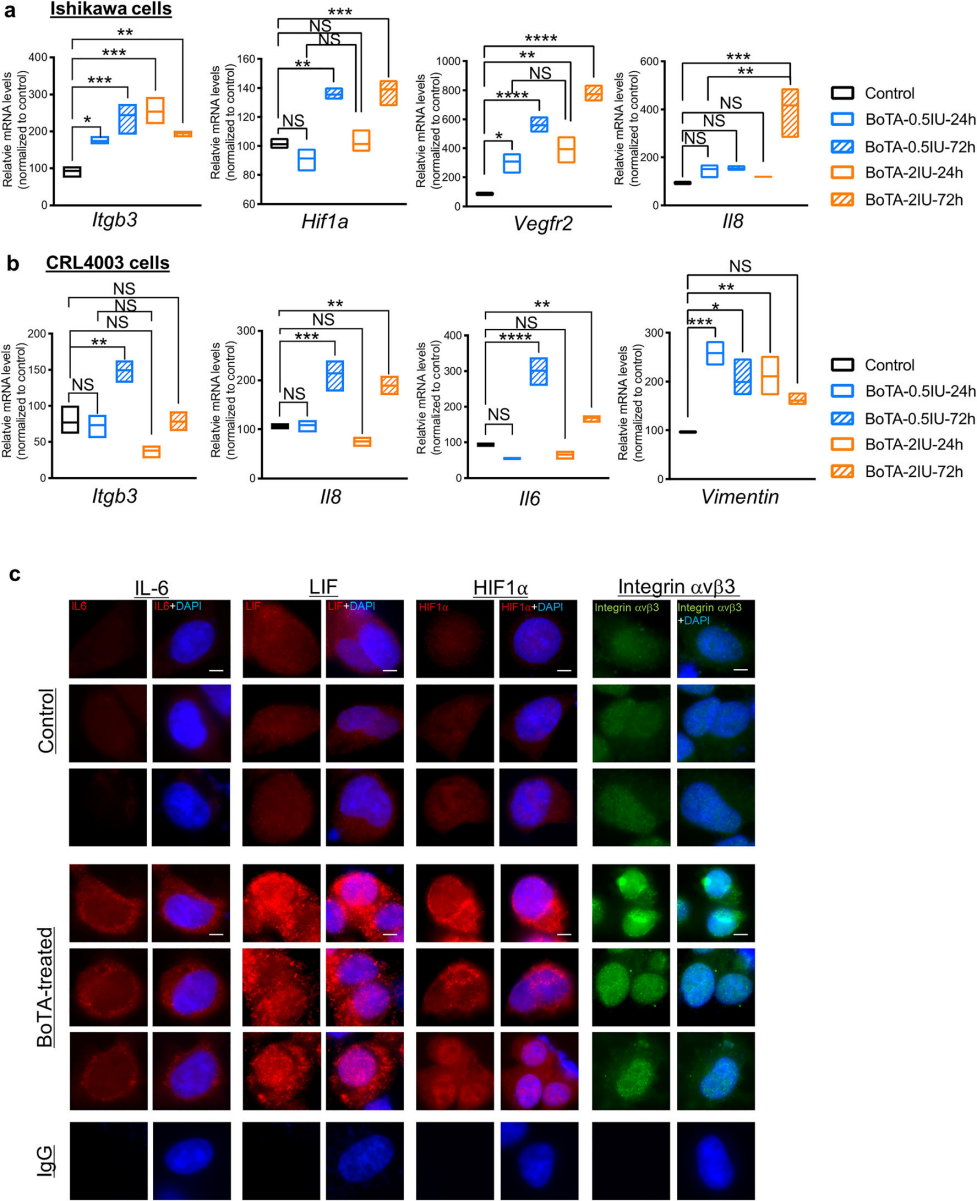
Endometrial receptivity-related factors are induced by BoTA treatment in endometrial cells. QRT-PCR analyses of *Itgb3*, *Hif1a*, *Vegfr2*, and *Il8* in endometrial epithelial (Ishikawa) cells (**a**), and *Itgb3*, *Il8*, *Il6*, and *Vimentin* in endometrial stromal (CRL4003) cells (**b**). (**c**) Immunofluorescence (IF) staining of IL-6, LIF, HIF1-⍺, and integrin ⍺vβ3 in Ishikawa cells in response to BoTA. Saline-treated cells were used for control. For the negative control, mouse or rabbit IgG was used. Scale bar: 20um

To investigate the effect of BoTA in the endometrial cells in regard to the regulation of endometrial receptivity, BoTA was applied to endometrial epithelial (Ishikawa) and stromal (CRL4003) cells *in vitro*. Quantitative RT-PCR analyses revealed that BoTA treatment induces significant increases of mRNA expression levels of endometrial receptivity-related genes including ITGB3, HIF1-α, VEGFR2, VIMENTIN, IL-6, and IL-8 in both Ishikawa and CRL4003 cells (Fig. 2a–b) suggesting both endometrial epithelial and stromal cells are responsive to BoTA treatment. Particularly, our data show that endometrial epithelial cells are more sensitive to BoTA treatment rather than stromal cells, which are evidenced by the findings showing that Ishikawa cells were more responsive to BoTA with a short-term treatment compared to CRL4003 cells. Even though both Ishikawa and CRL4003 immortalized cells might lack normal physiological features compared to primary endometrial cells, these data might implicate that endometrial epithelial layer is the more effective target of BoTA to induce the faster response of endometrial receptivity. Induction of endometrial receptivity-related genes was validated with immunofluorescence (IF) staining analyses, displaying higher expression of HIF1α, integrin αvβ3, and LIF in BoTA-treated group compared to control (Fig. 2c and Supplementary Fig. 1a–b). Of note, relatively higher concentration (10IU) of BoTA treatment rather led to reduction of all the expressions of endometrial receptivity-related markers (Supplementary Fig. 1c–d). Furthermore, to evaluate the toxicity or safety of BoTA treatment, we performed the cell-based *in vitro* assays to test if BoTA treatment induces cytotoxicity or aberrant cell proliferation in endometrial epithelial cells prior to further investigation for the clinical applicability of BoTA treatment. These analyses revealed that BoTA treatment induces impact on neither cytotoxicity nor aberrant cell proliferation in endometrial epithelial cells regardless of the concentration ranges between 0.5IU and 20IU (Supplementary Fig. 2a–b). Additionally, BoTA-induced migratory effects of endometrial cells were assessed. Fully confluent cells in the condition of starvation were scratched, and their migratory capacity was assessed by measuring the gap distance in between of two sides of cells (Supplementary Fig. 2c–d). No significant differences were observed in BoTA-treated (0.5 and 2.0IU) groups compared to controls indicating BoTA has no stimulatory effect on migratory capacity of both endometrial epithelial and stromal cells (Supplementary Fig. 2e–f). Our observations here demonstrate that BoTA treatment at an appropriate concentration increases endometrial receptivity-related gene expression in both endometrial epithelial and stromal cells *in vitro* implicating that BoTA treatment might induce positive regulatory effect of endometrial receptivity.

### Identification of Alterations in Gene Expression Induced by Intrauterine BoTA Infusion in Mice

**Fig. 3:**
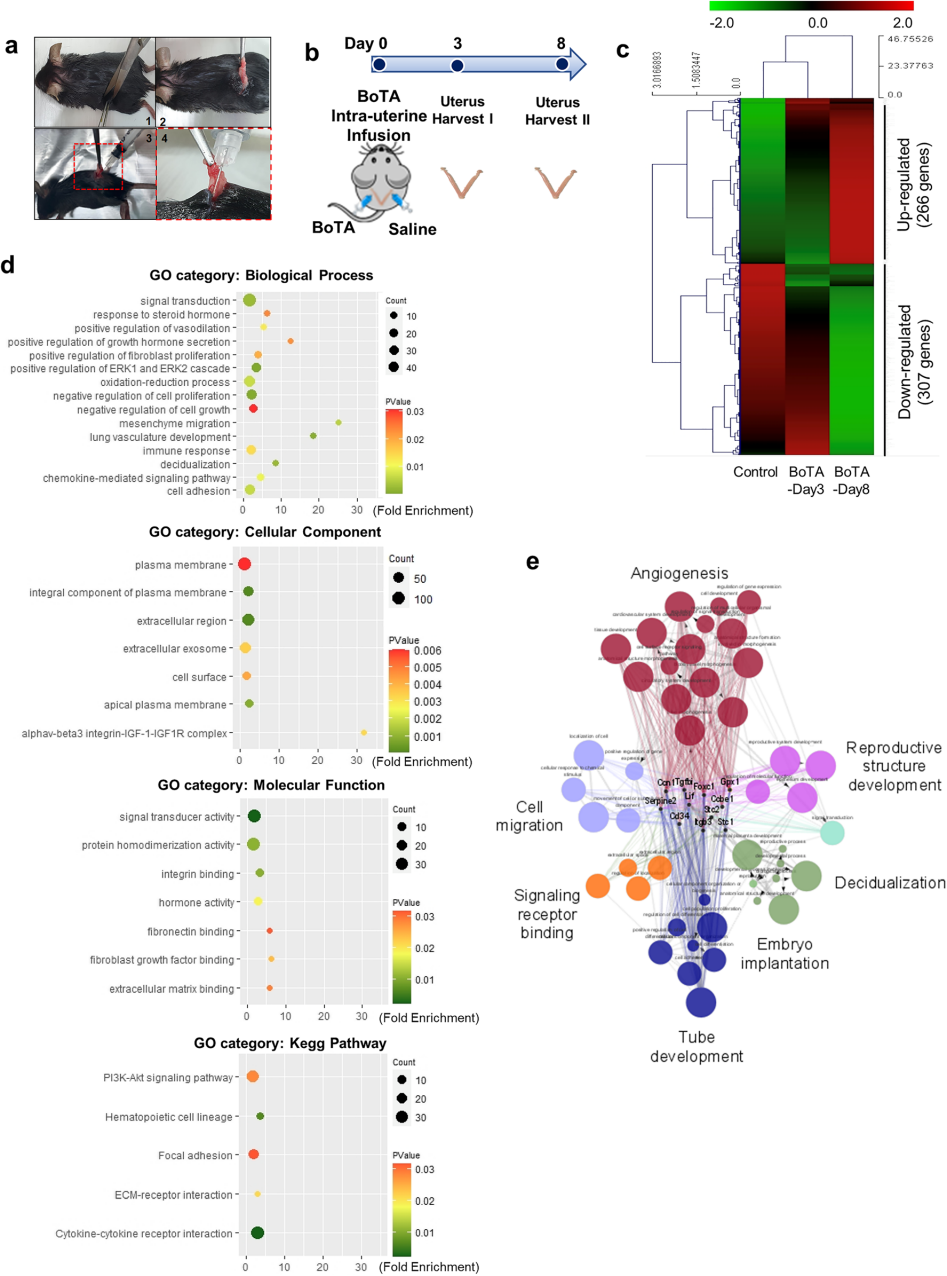
Identification of differentially expressed genes between the BoTA-treated uterus vs. control. (**a**) A procedure of intrauterine BoTA administration. (**b**) A schematic diagram of experimental plan of intrauterine BoTA administration. (**c**) Unsupervised hierarchical clustering analysis of RNA seq visualizing a heatmap plot showing differentially expressed genes (DEGs) in BoTA-treated group vs. control. Each row represents a distinct sample (5 independent BoTA-treated groups compared to saline-treated control), and each column represents an individual gene (a list of 23,282 genes). Normalized (log2) and standardized (each sample to mean signal=0 and standard deviation=1) level of gene expression is denoted by color (green, low; dark, intermediate; red, high), as indicated in the gradient panel. (**d**) Dot plots displaying gene ontology (GO) and pathway analysis of differentially expressed genes between BoTA-treated (Day 8) and control using DAVID tool and their fold enrichment on X-axis and gene count numbers on dot size. The cutoff for significance was set by *P* < 0.05. Biological process (BP), cellular component (CC), and molecular function (MF), KEGG pathway (KP) annotations. (**e**) A network of gene-gene interaction among BoTA-regulated genes was constructed displaying specifically enriched signaling pathways

To examine the *in vivo* effects of intrauterine application of BoTA, BoTA was infused into one side of mouse uterine horns, and saline was infused into the other side of horns for the control (Fig. 3a). Mouse uterine tissues were obtained 3 or 8 days after BoTA infusion, and total RNA was extracted from the whole endometrial tissues (Fig. 3b). In order to further examine the alterations of global gene expression depending on the intrauterine application of BoTA, RNA-seq data were generated from BoTA-treated versus saline-treated (control) endometrial tissues. Unsupervised hierarchical clustering analyses using a fold change cutoff of 2 and a *P*-value cutoff of 0.05 identified a total of 197 (88 genes were up-regulated and 109 genes were down-regulated) and 573 (266 genes were up-regulated and 307 genes were down-regulated) differentially expressed genes in BoTA-Day 3 and Day 8 groups, respectively (Fig. 3c and Supplementary Fig. 3a). To perform functional clustering of 573 differentially expressed genes, which were captured from Day 8 BoTA-treated uterine tissues, gene ontology (GO) and pathway analyses were performed by using the Database for Annotation, Visualization, and Integrated Discovery (DAVID) online tools [33]. Enriched GO terms in each category and pathway including associated gene counts, *P*-value, and FDR are shown in Table 1. The *P*-value and fold enrichment (FE)-value were calculated by Fisher’s exact test and multiple comparisons test, respectively (*P* < 0.05 and FE > 1.5). A total of 573 differentially expressed genes were classified according to GO terms, including biological process, BP; cellular component, CC; molecular function, MF; and KEGG pathway, KP (Fig. 3d and Table 1). These analyses demonstrate that specific BP categories, including response to steroid hormone (*P*=0.023429), positive regulation of growth hormone secretion (*P*=0.022572), decidualization (*P*=0.002337), positive regulation of vasodilation (*P*=0.012491), lung vasculature development (*P*=0.001022), and cell adhesion (*P*=0.006471), were enriched in BoTA-treated (Day 8) uterine tissues implicating biological processes particularly related to angiogenesis and the early phase of embryo-endometrial interaction in response to hormones. Moreover, enriched terms of the CC category include integral components of plasma membrane (*P*=4.39xE^-08^), apical plasma membrane (*P*=8.52xE^-04^), extracellular region (*P*=6.03xE^-14^), and cell surface (*P*=0.004213). Among the enriched CC components, integrin αvβ3-integrin IGF1-IGF1R complex (*P*=0.003231) is specifically reported to be strongly correlated to the early stage of embryo implantation [27]. Integrin binding (*P*=0.010497), signal transducer activity (*P*=0.002136), hormone activity (*P*=0.018643), fibroblast growth factor binding (*P*=0.023895), and extracellular matrix binding activity (*P*=0.029068) were composed of enriched MF terms. Pathway enrichment analyses based on KEGG pathway (KP) analyses were performed using either Chi-square test or Fisher’s exact test, revealing that pathways including cytokine-cytokine interaction (*P*=1.30xE^-04^) and PI3K-AKT signaling pathway (*P*=0.032839), hematopoietic cell lineage (*P*=0.005628), focal adhesion (*P*=0.037505), and ECM-receptor interaction (*P*=0.023927) were enriched in BoTA-treated (Day 8) uterine tissues (Fig. 3d and Table 1). These analyses implicate that intrauterine BoTA treatment might induce the intracellular signaling including ERK and PI3K-AKT pathway to possibly result in promoting angiogenesis [34, 35], which is supported by enrichment of signal transducer activity classified in BP and MF categories. Moreover, intercellular interactions might be enhanced in BoTA-treated endometrium, which was evidenced by high levels of responses including cytokine-cytokine receptor interaction (*P*=1.30xE^-04^), chemokine-mediated signaling pathway (*P*=0.009934), immune response (*P*=0.014198), and oxidation-reduction process (*P*=0.006635). Furthermore, in-depth clustering analyses revealed that BoTA treatment are strongly associated with the regulation of decidualization, hormonal activity, and interaction between surface adhesion molecules and ECM, which are critically correlated with endometrial receptivity, the process of embryo implantation, and maintenance of pregnancy [36]. Further analyses of enriched GO categories of Day 8 BoTA-treated endometrium compared to saline-treated group, visualized by ClueGO, revealed that BoTA-induced enrichment includes angiogenesis-related pathways such as angiogenesis, cell migration, signaling receptor binding, reproductive structure development, tube development, embryo implantation, and decidualization (Fig. 3e). Among these enrichments, 13 significantly differentially expressed genes including Ccl7, Cyr61, Itgb3, Foxc1, Clec14a, Hif3a, Gpx1, Cd34, Ccbe1, Tgfbi, Lif, Stc1, and Stc2 were specifically associated with GO terms of angiogenesis (GO:0001525) or embryo implantation (GO:0007566) (Table 2).

### Enhanced Endometrial Blood Vessel Formation by Intrauterine BoTA Treatment

**Fig. 4:**
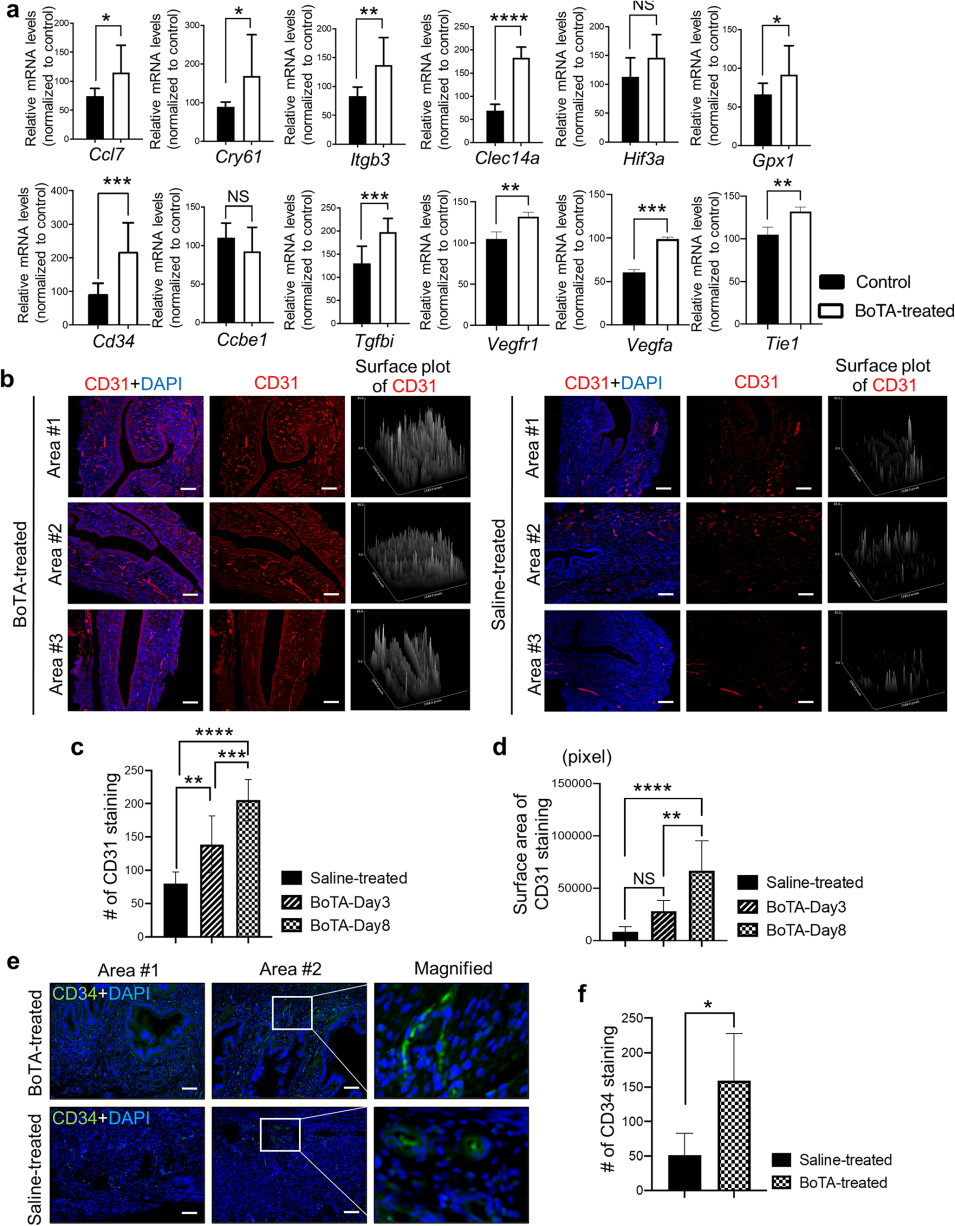
Induction of angiogenesis-related genes in BoTA-administered mouse uterus. (**a**) QRT-PCR analysis of *Ccl7*, *Cry61*, *Itgb3*, *Clec14a*, *Hif3a*, *Gpx1*, *Cd34*, *Ccbe1*, *Tgfbi*, *Vegfr1*, *Vegfa*, and *Tie1* in BoTA-treated uterus samples compared to controls. (**b**) IF staining of CD31 in longitudinally sectioned mouse uterus harvested 8 days after BoTA intrauterine infusion. Saline-treated endometrium was used for control. Scale bar: 100um. Representative two images from the different areas are shown in Supplementary Figure 4A. CD31 expression was quantified by counting the number of CD31 staining (**c**) and stained surface area (**d**). (**e**) IF staining of CD34 in longitudinally sectioned mouse uterus harvested 8 days after BoTA intrauterine infusion. Saline-treated endometrium was used for control. Scale bar: 100um. CD34 expression was quantified by counting the number of CD34 staining (**f**). Comparison groups for (**a**) and (**f**) are from 3 independent experiments and analyzed using the unpaired Student *t*-test for parametric distributions and the multiple comparisons for (c) and (d) are from 3 independent experiments and analyzed using the ordinary one-way ANOVA analysis with Dunnett’s multiple comparison test including *P*-values (*<0.05, **<0.01, ***<0.001, ****<0.0001, *NS* not significant)

In order to validate our RNA-seq data, genes that are classified and strongly related to angiogenesis including *Ccl7*, *Cry61*, *Itgb3*, *Clec14a*, *Hif3a*, *Gpx1*, *Cd34*, *Ccbe1*, *Tgfbi*, *Vegfr1*, *Vegfa*, and *Tie1* were subjected to quantitative QRT-PCR analyses. The validation revealed that the expression pattern examined by QRT-PCR was concordant with RNA-seq data (Fig. 4a). To further corroborate the evidence for the induction of endometrial angiogenesis with BoTA infusion in mouse uterus, our interrogation of paraffin-embedded BoTA-treated endometrial tissue sections with IF staining of CD31, a surrogate marker for blood vessel formation, revealed that BoTA treatment increased CD31 expression in mouse uterus compared to control group (Fig. 4b, supplementary Fig. 4a–b), which is consistent with our RNA-seq analyses. In particular, BoTA-infused endometrial tissues exhibited enhanced CD31 levels in the stromal layer especially close to the lining of epithelium. Moreover, the total number and surface area of CD31 staining were further quantified exhibiting significant and gradual increase in BoTA-treated groups (Day 3 and 8) compared to control (Fig. 4c–d). As a novel marker for angiogenesis of vascular endothelial cells and progenitor cells with stemness [37], CD34 expression pattern was assessed in paraffin-embedded BoTA-treated endometrial tissue sections revealing more abundantly expressed CD34 in BoTA-treated groups compared to control (Fig. 4e–f, Supplementary Fig. 4b). These observations indicate that BoTA infusion might have an angiogenic effect to enhance the development of new capillaries from pre-existing blood vessels in the endometrium.

### Improved Endometrial Receptivity by Intrauterine BoTA Treatment

**Fig. 5:**
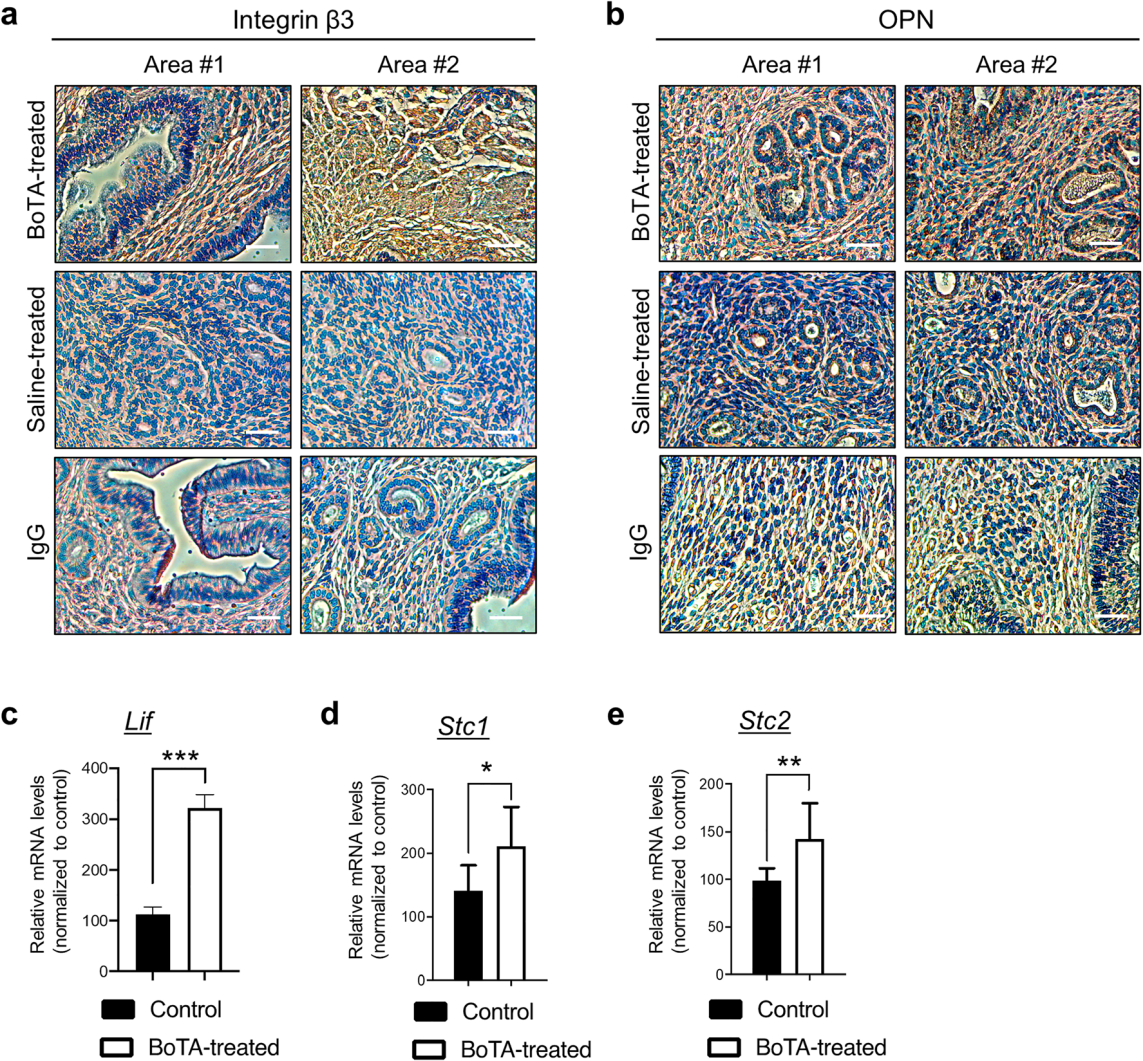
Induction of endometrial receptivity-related genes in BoTA-administered mouse uterus. Representative two images of immunohistochemistry analyses of integrin β3 (**a**) and OPN (**b**) in longitudinally sectioned mouse uterus harvested 8 days after BoTA intrauterine infusion. Saline-treated endometrium was used for control. For the negative control, mouse or rabbit IgG was used. Scale bar: 20um. QRT-PCR analysis of *Lif* (**c**), *Stc1* (**d**), and *Stc2* (**e**) in BoTA-treated uterus samples compared to controls. Comparison groups for (**c**–**e**) are from 3 independent experiments and analyzed using the unpaired Student *t*-test for parametric distributions including *P*-values (*<0.05, **<0.01, ***<0.001)

Among significantly altered embryo implantation-related genes, differential expression pattern of *Itgb3* and *Lif* upon BoTA treatment showed consistency with data from *in vitro* analyses shown in Fig. 2. In order to further support our RNA-seq data, genes that are classified and strongly related to embryo implantation, including integrin β3, osteopontin (OPN), LIF, STC1, and STC2, were subjected to immunohistochemistry or QRT-PCR analyses. Integrin β3 and its ligand OPN proteins were remarkably highly expressed in BoTA-treated endometrial tissue section compared to saline-treated control group (Fig. 5a–b). The validation with QRT-PCR revealed concordant pattern of expression of *Lif*, *Stc1*, and *Stc2* with those from RNA-seq analyses (Fig. 5c–e), suggesting that intrauterine infusion of BoTA might be an effective method to improve the endometrial receptivity for patients who are suffering from implantation failure with poor receptivity.

### BoTA-Treated Uterine Fertility Assessment

**Fig. 6:**
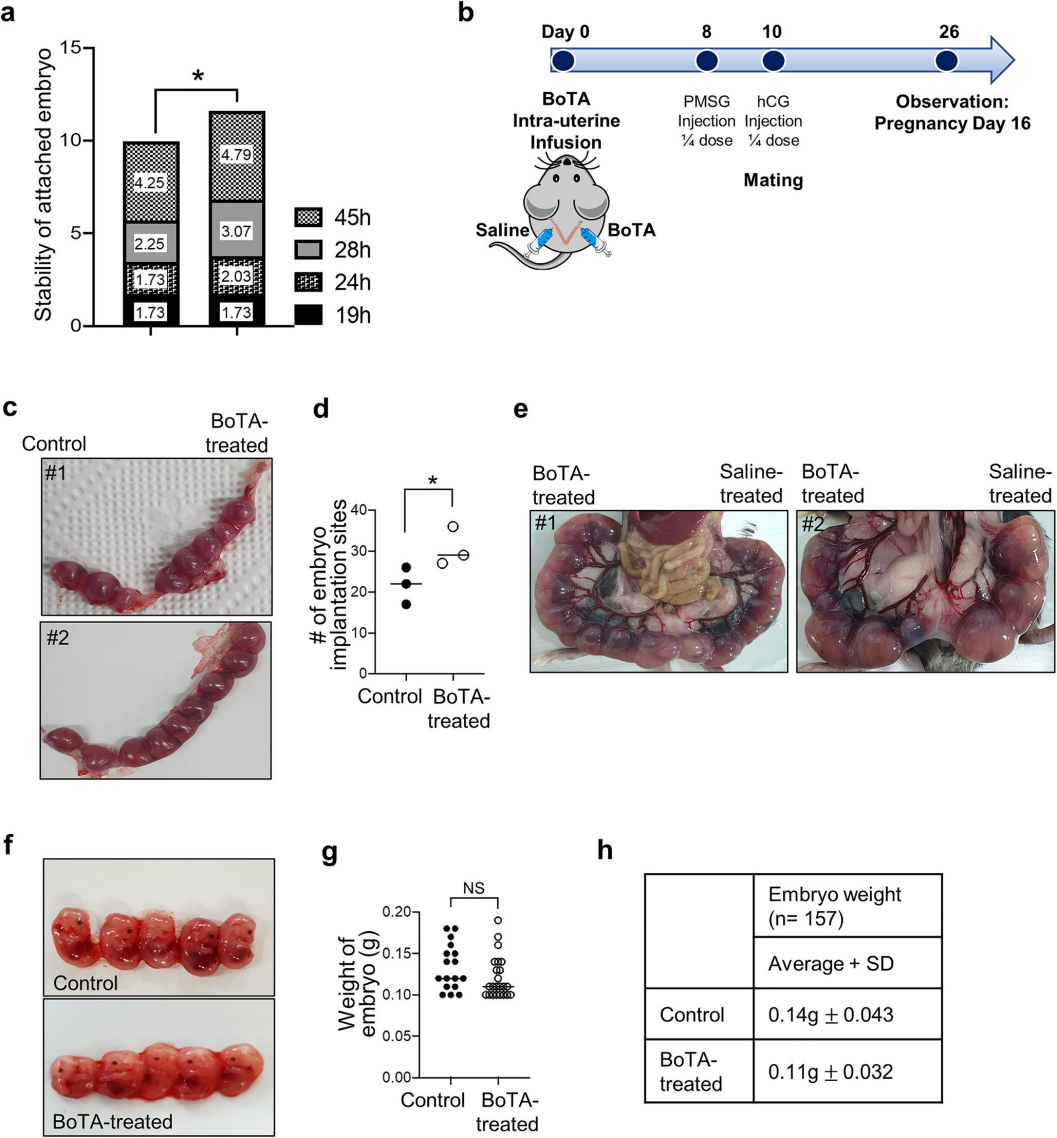
The impact of intrauterine administration of BoTA on fertility assessment. (**a**) Rates of stability of attached mouse embryos onto saline- or primed BoTA-treated Ishikawa cells at 19h, 24h, 28h, or 45h of co-culture. (**b**) A procedure of intrauterine BoTA administration. (**c**) Representative two images of mouse uterus including embryo implantation sites. (**d**) Average numbers of embryo implantation sites from 34 mouse uterus horns were assessed on embryonic day 16. (**e**) Representative images of mouse uterine arteries towards the sites of embryo implantation after open surgery. (**f**) Representative images of embryos (day 16) obtained from BoTA-treated or saline-treated mouse uterus. (**g**–**h**) Average weight of embryos obtained from BoTA-treated uterus compared to control. Data shown for (**d**) and (**g**) are from 3 independent experiments (# of mice, 17; # of embryos, 157) and analyzed using the paired Student *t*-test for parametric distributions including *P*-values (*<0.05, **<0.01, ***<0.001, *NS* not significant).

Angiogenesis is known to participate in a wide range of the process in pregnancy including folliculogenesis, early implantation, placentation, and embryonic development [2, 38]. This led us to examine the correlation of enhanced neovascular activity and increased molecular features of endometrial receptivity induced by BoTA treatment with the outcomes of embryo implantation and pregnancy. For the assessment of BoTA effect on the stability of embryo attachment *in vitro*, a total of 73 day 5 mouse embryos were transferred (one embryo per well) to confluent Ishikawa cells, which were treated with BoTA (primed; 5h of exposure to BoTA prior to embryo co-culture, non-primed; commence BoTA exposure at the time of embryo co-culture) and co-cultured for 19h, 24h, 28h, or 45h. The stability of attached mouse embryo was assessed according to the 5-stage standard: score 1, floating; 2, weakly attached but detached after tapping; 3, weakly attached but stuck at the attachment site after tapping; 4, stably attached; and 5, stably attached and showed outgrowth [29, 30]. Up to 24h of co-culture, embryos transferred onto both control and BoTA-treated cells showed no significant difference in their attachment stability. However, at the late stage of co-culture (24–48h), embryos were more stably attached onto BoTA-primed treated Ishikawa cells compared to controls (*P*=0.0332) (Fig. 6a). Of particular note, no significant difference was observed in the stability of attached embryo onto non-primed BoTA-treated Ishikawa cells compared to that of control cells (*P*=0.6322) (Supplementary Fig. 5a), suggesting an effective time-point for BoTA treatment to improve the rates of embryo implantation, which should be primed for the embryo transfer onto endometrial epithelial cells. In order to verify the efficacy of BoTA *in vivo*, BoTA was applied to mouse uterus in a same manner as shown in Fig. 3a. After 8 days of BoTA infusion, both sides of uterine horns were obtained to expose the embryo implantation sites 16 days after mating (Fig. 6b). These analyses revealed that the total number of implantation sites in BoTA-treated group was significantly higher than controls (*P*=0.0121) (Fig. 6c–d). Intriguingly, increased numbers and thickness of uterine arteries, which formed the network towards each site of embryo implantation in mouse uterus, were observed at the open abdominal surgery in BoTA-administered side compared to control (Fig. 6e). This might be because of increased endometrial neomicrovessles, which might have been developed and stimulated from pre-existing uterine arteries, induced by BoTA intrauterine treatment. Importantly, no retarded embryos were detected in both groups, and the weight of embryos from both groups showed no significant difference (Fig. 6f–h). Representative images of the implantation sites in the uterus of mice and embryos obtained from different conditions are shown in Fig. 6c and f.

## Discussion

In this current study, we demonstrate that the intrauterine BoTA administration significantly induces endometrial angiogenesis displaying increased numbers of vessel formation, which is consistent with the findings of increased tube formation and sprouting area of endothelial cells *in vitro*. Furthermore, BoTA administration enhances the endometrial receptivity and subsequently improves the rates of embryo implantation with no morphologically retarded embryos. Even though the molecular and cellular mechanisms underlying BoTA-induced endometrial angiogenesis and its direct or indirect correlation with embryo implantation remains to be defined, this might suggest intrauterine application of BoTA, which is totally non-invasive causing no traumatic injury to the endometrium at all, as an effective therapeutic strategy for patients who are suffering from repeated implantation failure with the problems with endometrial receptivity.

Endometrial angiogenesis is thought to be fundamental in supporting endometrial growth and repair that provide vascularized endometrial receptivity, which usually refers to the ability of the endometrial lining to accept and accommodate a nascent embryo [38, 39]. To support this notion, it has been previously reported that increased endometrial blood flow is associated with the pregnancy outcome during the time of window of implantation [40]. This can be evidenced by our observations displaying the enhanced capacity of endothelial cell tube formation and sprouting towards the endometrial stromal cell layer with BoTA treatment, implicating the impact of BoTA on the increased vascular network and blood supply to the endometrial lining, which might provide a better microenvironment for the embryo implantation. This is consistent to the previous findings from reconstructive and aesthetic surgery fields evaluating the angiogenic effect of BoTA application demonstrating increased endothelial cell sprouting with higher numbers of vessel formation *in vitro* [26] and elevated transverse rectus abdominis myocutaneous flap survival by BoTA-induced angiogenic effect via increased HIF1α/VEGF signaling [25]. Significant induction of endometrial receptivity-related genes both in cultured endometrial epithelial or stromal cells with BoTA treatment indicates that BoTA induces the expression of those genes even in the independent and separate culture condition of endometrial epithelium or stroma (Fig. 2). Moreover, the positive regulation of BoTA application in the endometrial angiogenesis and receptivity was strongly evidenced by the comparison of RNA-seq of BoTA-treated mouse uterus tissues to saline-treated controls. Thirteen significantly differentially expressed genes, including CCL7, CYR61, ITGB3, FOXC1, HIF3A, GPX1, CD34, CCBE1, TGFBI, LIF, STC1, and STC2, were sorted for GO terms of angiogenesis or embryo implantation. It has been reported that CCL7, CYR61, FOXC1, and CD34 play crucial roles in vascular remodeling and endothelial cell proliferation [41–44]. Additionally, LIF is known to be strongly related to both angiogenesis and embryo implantation via activation of JAK/STAT and MAPK signaling [45]. STC1 and STC2 have been suggested as key players displaying increased expression levels at the sites of embryo implantation [46]. This has been dramatically supported by the data showing increased levels of CD31 and CD34 expression in intrauterine BoTA-treated mouse endometrial lining compared to control; effects of BoTA in vessel formation were assessed by measuring the total numbers of staining and quantification of stained surface area (Fig. 4b–f). Furthermore, the pregnancy outcomes revealing significantly higher rates of embryo implantation in BoTA-treated horn of mouse uterus compared to the rates in saline-treated uterine horn support RNA-seq analyses (Figs. 4 and 6). However, high concentration of BoTA application restores or even reduces the levels of angiogenesis and endometrial receptivity-related gene expression back to the levels observed in control groups (Supplementary Fig. 1), which is consistent to the previous findings showing the interference of angiogenesis by high concentrations of BoTA treatment in endothelial cells *in vitro* [26]. This might implicate that the dose selection and adjustment might be a critical process for the clinical trial to patients with endometrial problems.

The effect of BoTA on the microvascular remodeling and its activation has been studied to a lesser extent. Consistent with the previous reports demonstrating a significant increase in resting diameter of arterioles and venules with the topical application of BoTA to the rat cremaster muscle [24, 47], in the present study, we have demonstrated that non-invasive intrauterine BoTA administration increases the numbers of microvessel formation in the lining of the endometrium, which has been shown with the assessment of CD31 and CD34 immunoreactivity in the longitudinally sectioned tissues (Fig. 4), and more abundant and thicker uterine arteries were observed surrounding the sites of embryo implantation in the BoTA-applied mouse uterus compared to controls (Fig. 6g). Even though the detailed mechanisms of these actions are still required to be elucidated in the future study, these data might imply that absorbed BoTA from the endometrial surface by the non-invasive intrauterine administration stimulates the formation of neovessels or elongation of existing microvessels towards the innermost lining of the endometrium and also induces the formation of uterine arteries outside the uterus probably to provide better and more fertile environment to the sites of embryo implantation.

A variety of studies evaluated the impact of BoTA on temporary muscular paralysis and relief of the tension on the sites of wound, which may aid to prevent the hypertrophy and hyperpigmentation of wound sites [48, 49]. BoTA is also widely investigated and applied in pain management of myofascial syndrome, headaches, arthritis, and neuropathic pain by induction of muscle chemodenervation via preventing the release of neurotransmitters such as acetylcholine and noradrenaline [50, 51]. A broad range of all these BoTA utilization may affirm its safety in human body, which has been addressed in the previous studies by demonstrating the remarkably effective and safe results of BoTA treatment for the facial aesthetic purposes, obtained from 1474 subjects [52], and quantitatively safe and tolerable profiles of BoTA utilization from the meta-analysis involving 2309 subjects [53]. Consistent with these reports, in our current study, we revealed that BoTA induces neither cytotoxicity, aberrant cell proliferation, nor irregular migratory ability (Supplementary Fig. 2). Moreover, there was no significant difference observed in the weight of day 16 embryos between BoTA-treated and saline-treated groups, especially no retarded embryos were observed in any groups (Fig. 6f–h), suggesting non-invasive intrauterine administration of BoTA as an effective therapeutic strategy with the safety.

Significantly, we demonstrated that increased numbers of microvessels were formed in the mouse endometrium with intrauterine BoTA treatment accompanied with elevated levels of angiogenesis-related marker expression. Additionally, enhanced rates of embryo implantation without any retarded embryos were observed in BoTA-treated mice compared to control group. Taken together, we provided a novel evidence suggesting an intrauterine BoTA administration as an effective therapeutic intervention to improve the endometrial microenvironment by increasing endometrial blood supply. Further study will be required to uncover the molecular mechanisms underlying the induction of angiogenesis by BoTA and direct or indirect association between BoTA-induced endometrial angiogenesis and the embryo implantation. Moreover, it will be essential to evaluate the reproductive toxicity of BoTA intrauterine application prior to clinical trials. A better understanding of uterine endometrial angiogenesis induced by BoTA may aid to develop a non-invasive novel method for clinical treatment of repeated implantation failure patients with less risk.

## Supplementary Information

Supplementary Figure 1. **Effect of BoTA in endometrial cells**. Immunofluorescence (IF) staining of LIF **(a)** and HIF1-⍺ **(b)** in Ishikawa cells in response to BoTA (0.5IU). The intensity of LIF and HIF1-⍺ expression in each image was further quantified by surface profiling. Saline-treated cells were used for control. Scale Bar; 20um. QRT-PCR analysis of *Vegfr2* and *Hif1a* in BoTA-treated (0.5IU-10IU) endometrial epithelial (Ishikawa) cells **(c)**, and *Itgb3* and *Vimentin* in endometrial stromal (CRL4003) cells **(d)**. (PNG 4948 kb).

High resolution image (TIF 2352 kb).

Supplementary Figure 2. **No cytotoxic effect of BoTA on endometrial epithelial cells** ***in vitro***. Effect of BoTA on cytotoxicity **(a)** measured by viability of Ishikawa cells (BoTA 0.5IU-20IU) and cell proliferation **(b)** measured by differences in cell number counting. Wound healing assay at 0h and 48h time point after scratch in BoTA-treated vs. saline-treated Ishikawa **(c)** and CRL4003 cells **(d)**, and wound closure was assessed by measuring the distance of gap **(e-f)**. (PNG 4948 kb).

High resolution image (TIF 6221 kb).

Supplementary Figure 3. **Identification of differentially expressed genes of the BoTA-treated uterus vs. control**, **(a)** A volcano plot displaying the comparison of differentially expressed genes (DEGs) between the BoTA-treat (Day 3) and control groups. The red dots indicate up-regulated DEGs, green dots indicate down-regulated DEGs, and blue dots indicate no DEGs between the BoTA-treated (Day 3) and untreated samples. Marked genes are associated with angiogenesis and embryo implantation. DEGs are selected by cut off values of fold change > 2 and p-value < 0.05 to identify significantly differentially expressed genes.. (PNG 4948 kb).

High resolution image (TIF 726 kb).

Supplementary Figure 4. **CD31 induction in BoTA-treated mouse uterus**, **(a)** Representative two images of IF staining of CD31 in longitudinally-sectioned mouse uterus harvested 8 days after BoTA intrauterine infusion. Saline-treated endometrium were used for control. Scale Bar; 100um. **(b)** Representative two images of IF staining of negative control for CD31 and CD34. (PNG 4948 kb).

High resolution image (TIF 1810 kb).

Supplementary Figure 5. **Embryo attachment in non-primed BoTA-treated Ishikawa cells**, **(a)** Rates of stability of attached mouse embryos onto saline- or non-primed BoTA-treated Ishikawa cells at 19h, 24h, 28h, or 45h of co-culture. (PNG 4948 kb).

High resolution image (TIF 537 kb).

## Data Availability

RNA-seq data that support the findings of this study have been deposited in GEO with the primary accession code GSE146934. The authors declare that all other data supporting the findings of this study are available within the article and its Supplementary information files.
