# Supplementary figures and images for: Non-invasive Intrauterine Administration of Botulinum Toxin A Enhances Endometrial Angiogenesis and Improves the Rates of Embryo Implantation
Source: Reprod Sci. 2021 Mar 1;28(6):1671–87. doi: 10.1007/s43032-021-00496-4 (PMC8144131; doi:10.1007/s43032-021-00496-4)

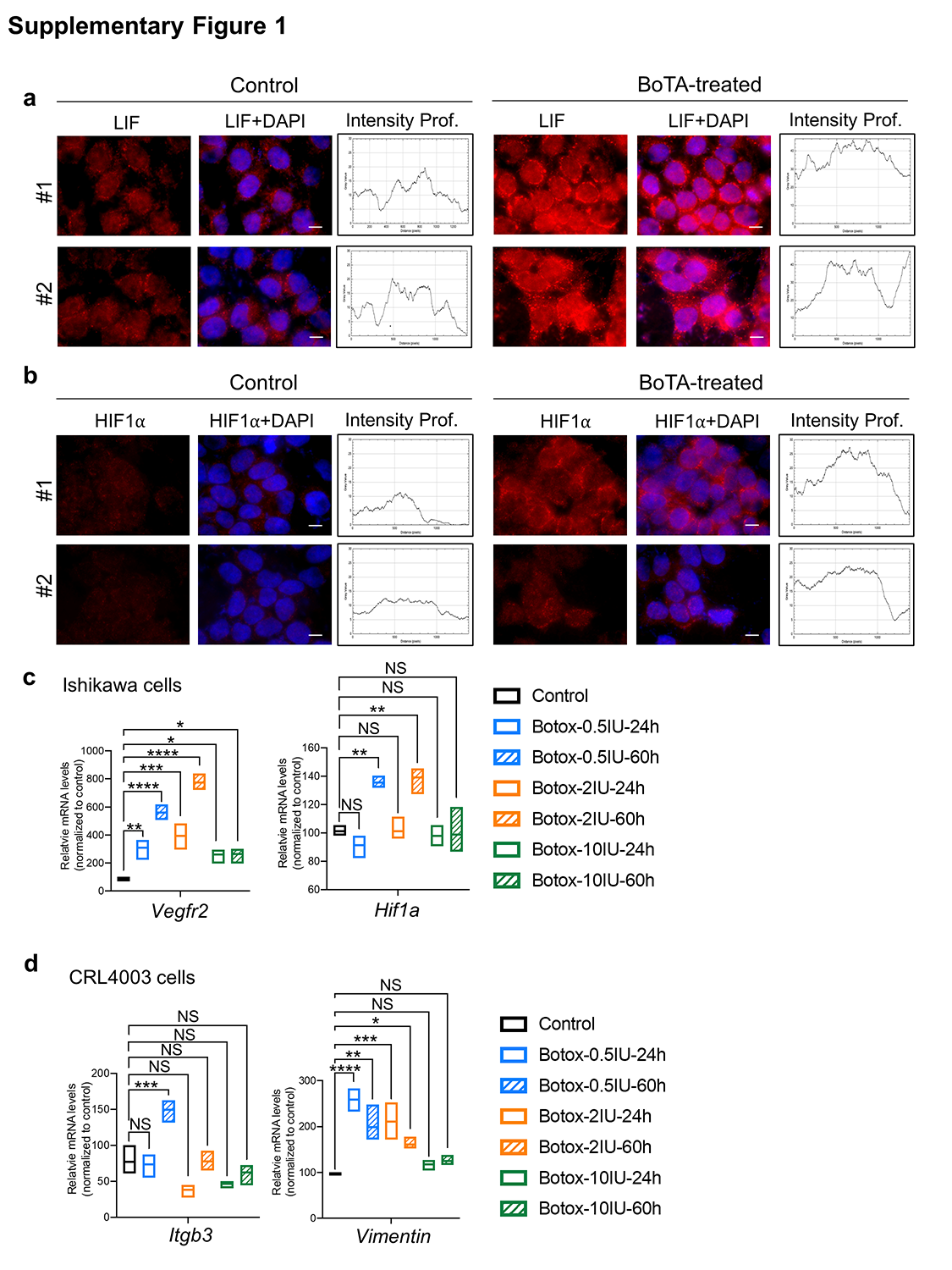

Supplement: Supplementary file 1 — Effect of BoTA in endometrial cells. Immunofluorescence (IF) staining of LIF (a) and HIF1-⍺ (b) in Ishikawa cells in response to BoTA (0.5IU). The intensity of LIF and HIF1-⍺ expression in each image was further quantified by surface profiling. Saline-treated cells were used for control. Scale Bar; 20um. QRT-PCR analysis of Vegfr2 and Hif1a in BoTA-treated (0.5IU-10IU) endometrial epithelial (Ishikawa) cells (c), and Itgb3 and Vimentin in endometrial stromal (CRL4003) cells (d). (PNG 4948 kb). [file 43032_2021_496_Fig7_ESM.png]

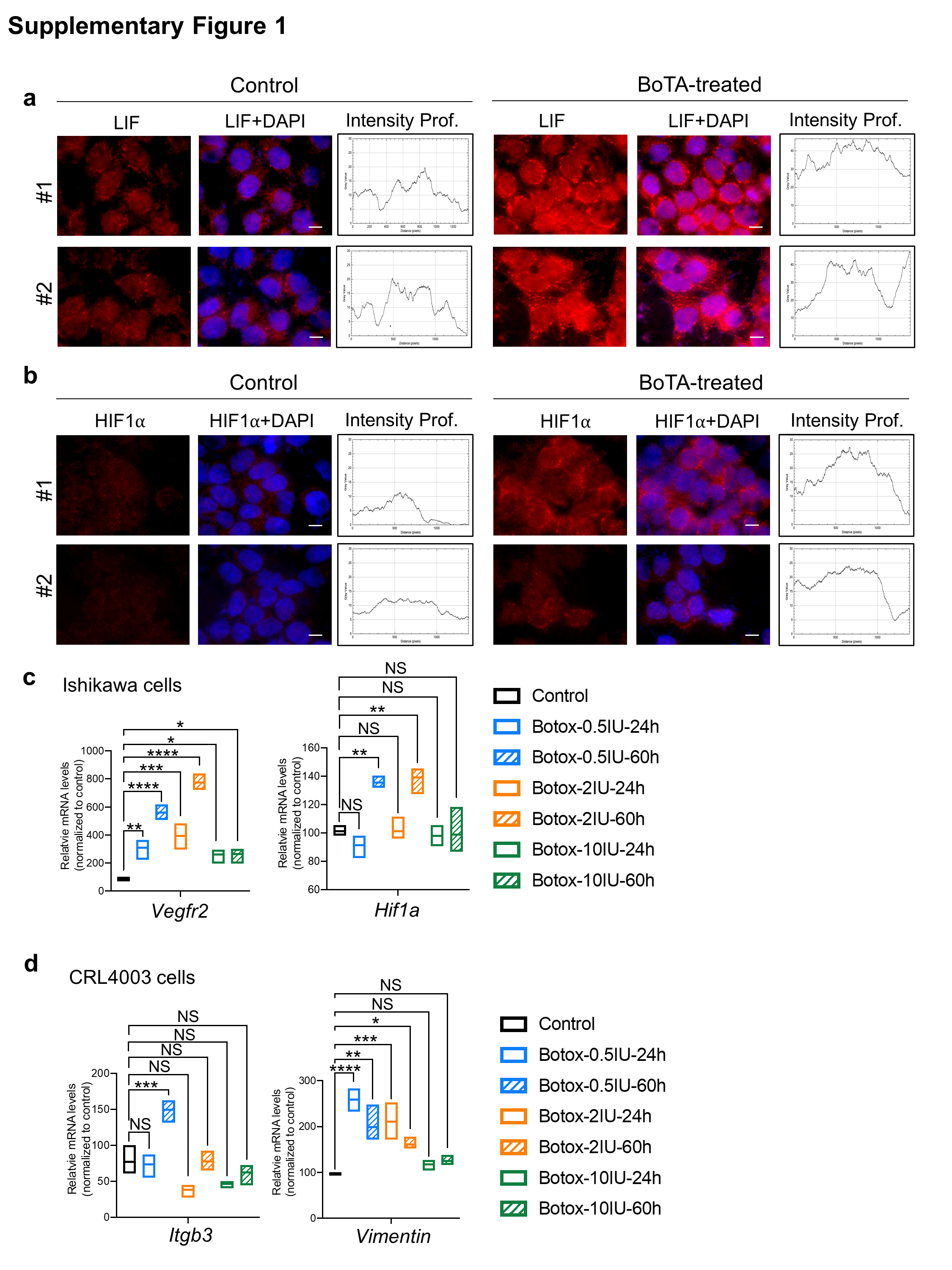

Supplement: Supplementary file 2 — High resolution image (TIF 2352 kb). [file 43032_2021_496_MOESM1_ESM.tif]

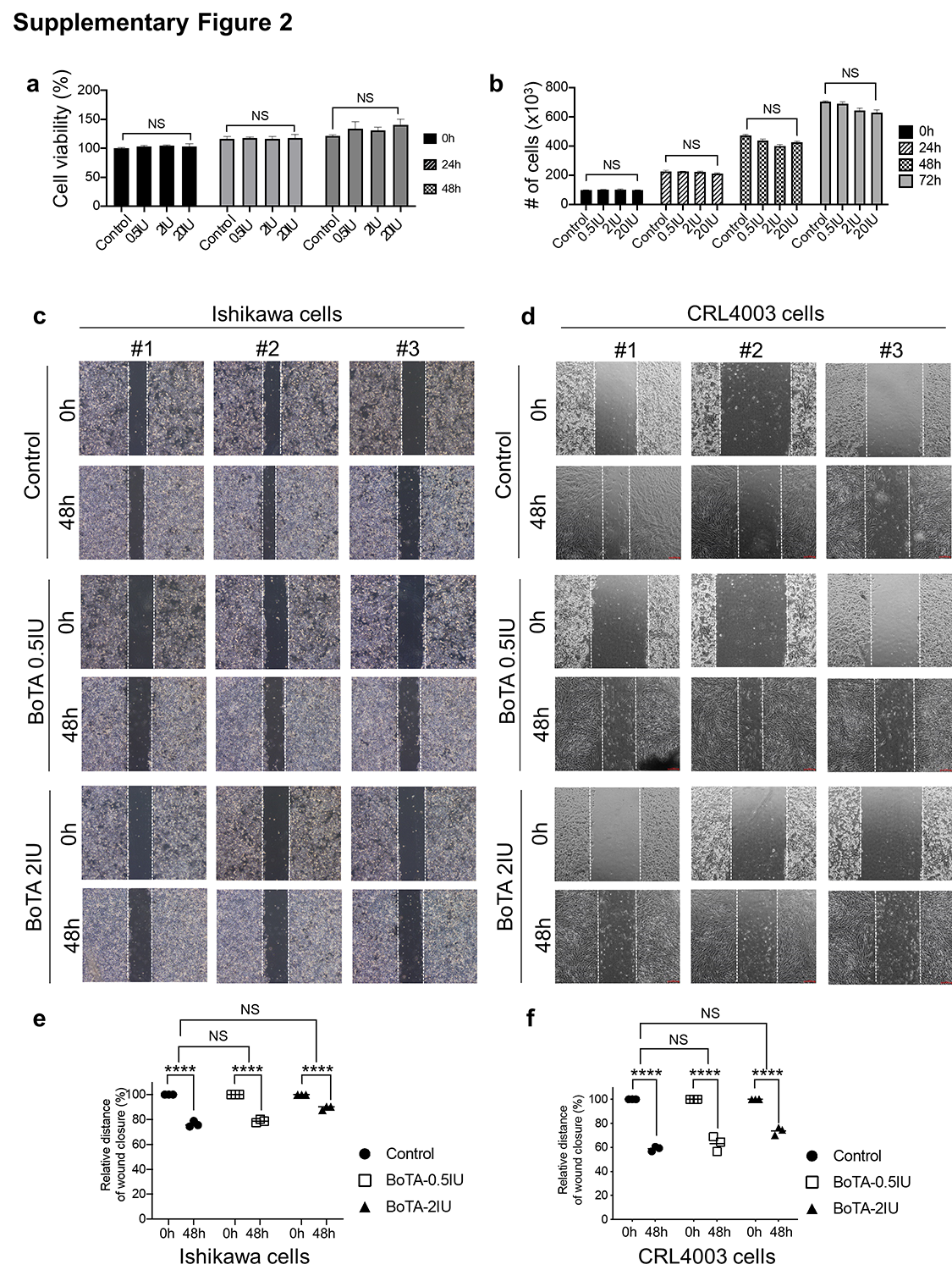

Supplement: Supplementary file 3 — No cytotoxic effect of BoTA on endometrial epithelial cells in vitro. Effect of BoTA on cytotoxicity (a) measured by viability of Ishikawa cells (BoTA 0.5IU-20IU) and cell proliferation (b) measured by differences in cell number counting. Wound healing assay at 0h and 48h time point after scratch in BoTA-treated vs. saline-treated Ishikawa (c) and CRL4003 cells (d), and wound closure was assessed by measuring the distance of gap (e-f). (PNG 4948 kb). [file 43032_2021_496_Fig8_ESM.png]

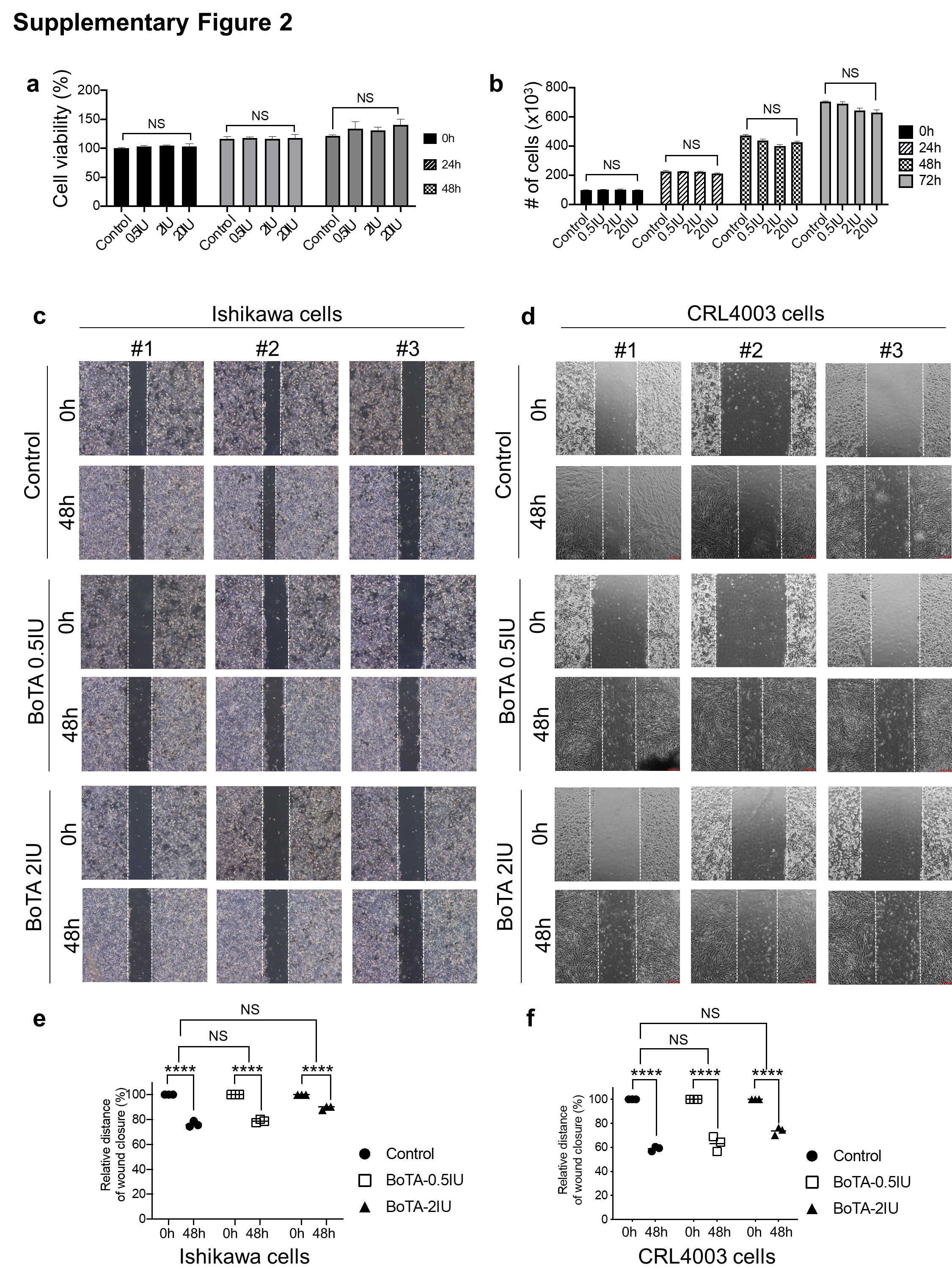

Supplement: Supplementary file 4 — High resolution image (TIF 6221 kb). [file 43032_2021_496_MOESM2_ESM.tif]

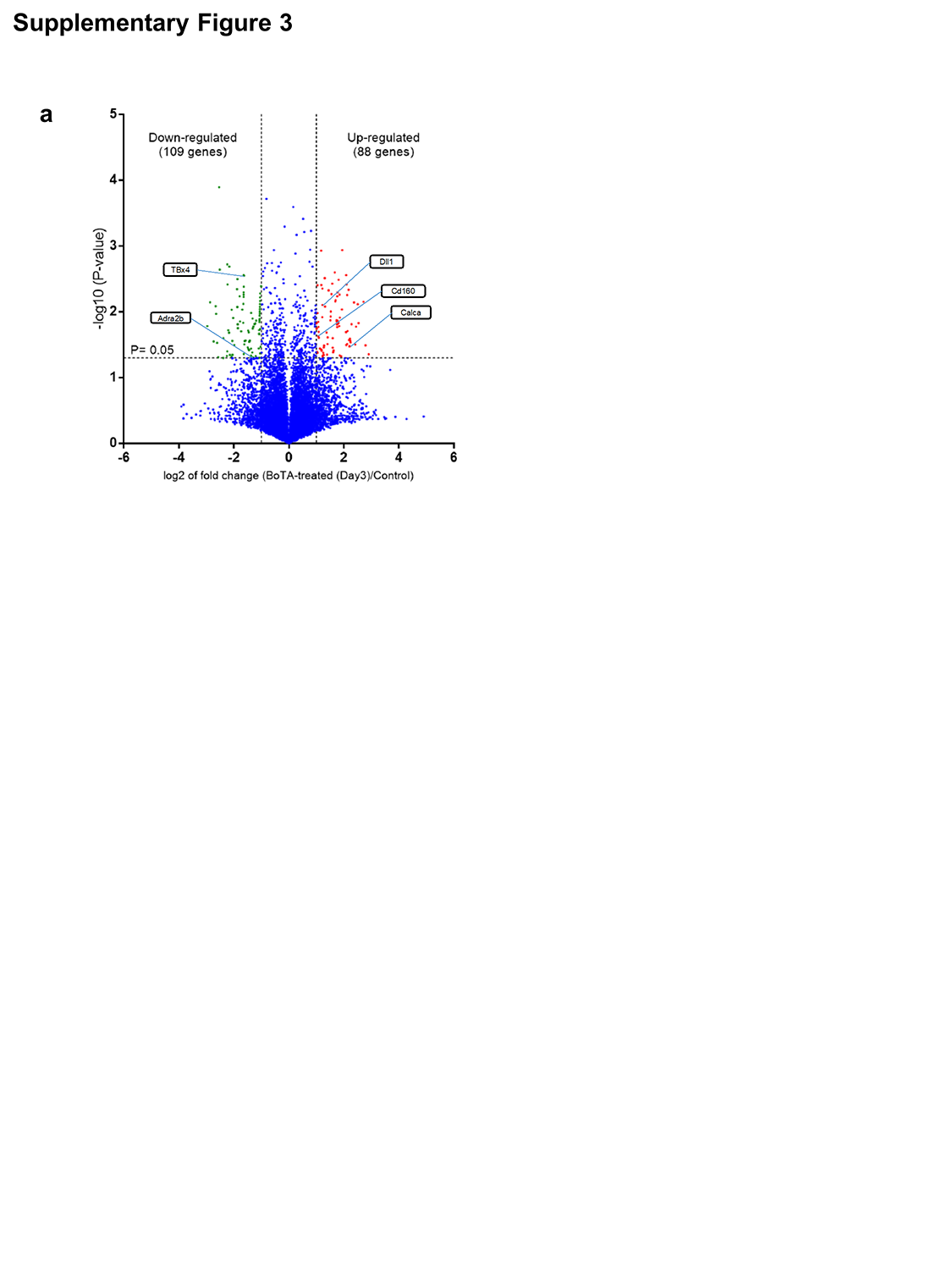

Supplement: Supplementary file 5 — Identification of differentially expressed genes of the BoTA-treated uterus vs. control, (a) A volcano plot displaying the comparison of differentially expressed genes (DEGs) between the BoTA-treat (Day 3) and control groups. The red dots indicate up-regulated DEGs, green dots indicate down-regulated DEGs, and blue dots indicate no DEGs between the BoTA-treated (Day 3) and untreated samples. Marked genes are associated with angiogenesis and embryo implantation. DEGs are selected by cut off values of fold change > 2 and p-value < 0.05 to identify significantly differentially expressed genes.. (PNG 4948 kb). [file 43032_2021_496_Fig9_ESM.png]

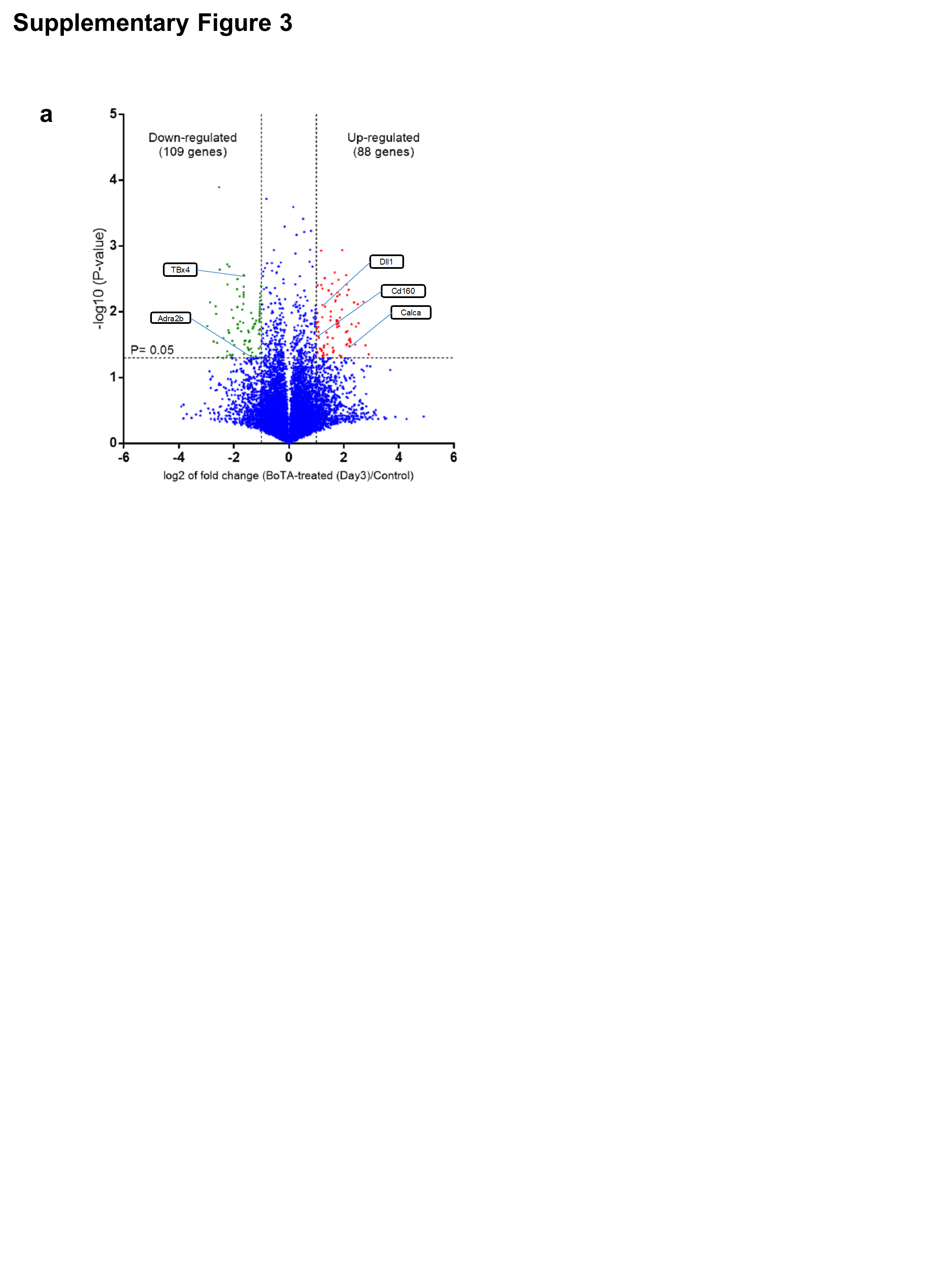

Supplement: Supplementary file 6 — High resolution image (TIF 726 kb). [file 43032_2021_496_MOESM3_ESM.tif]

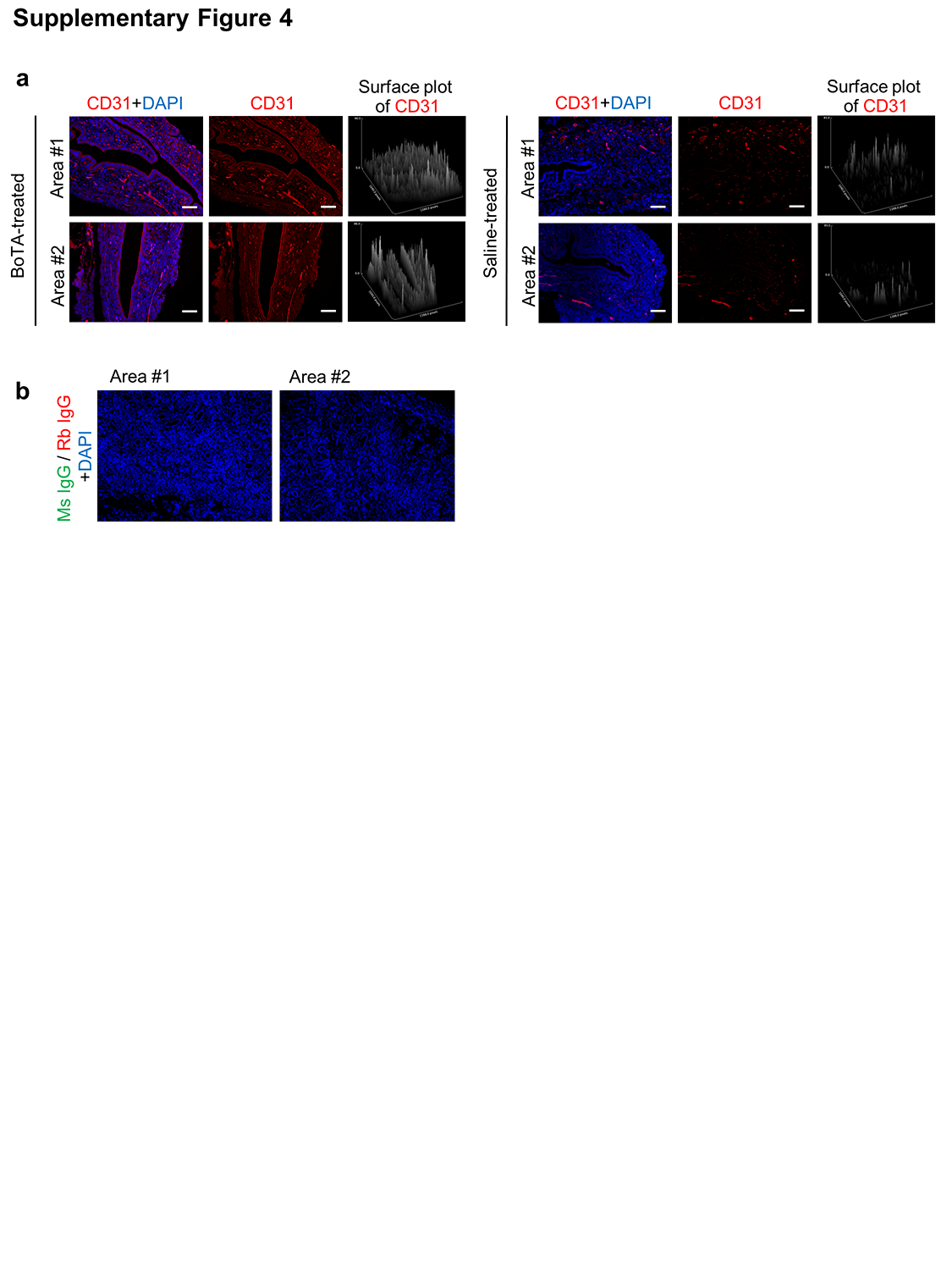

Supplement: Supplementary file 7 — CD31 induction in BoTA-treated mouse uterus, (a) Representative two images of IF staining of CD31 in longitudinally-sectioned mouse uterus harvested 8 days after BoTA intrauterine infusion. Saline-treated endometrium were used for control. Scale Bar; 100um. (b) Representative two images of IF staining of negative control for CD31 and CD34. (PNG 4948 kb). [file 43032_2021_496_Fig10_ESM.png]

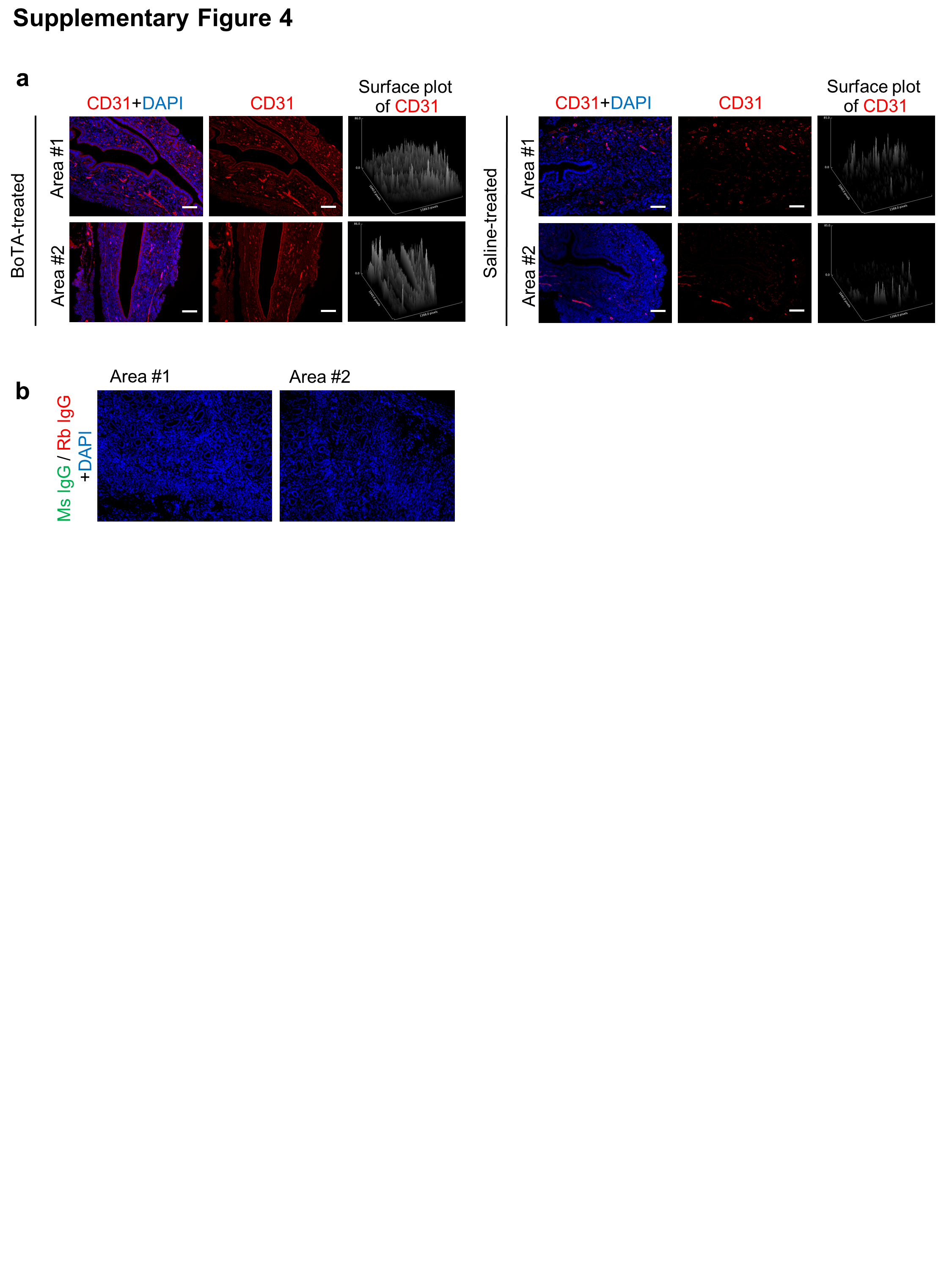

Supplement: Supplementary file 8 — High resolution image (TIF 1810 kb). [file 43032_2021_496_MOESM4_ESM.tif]

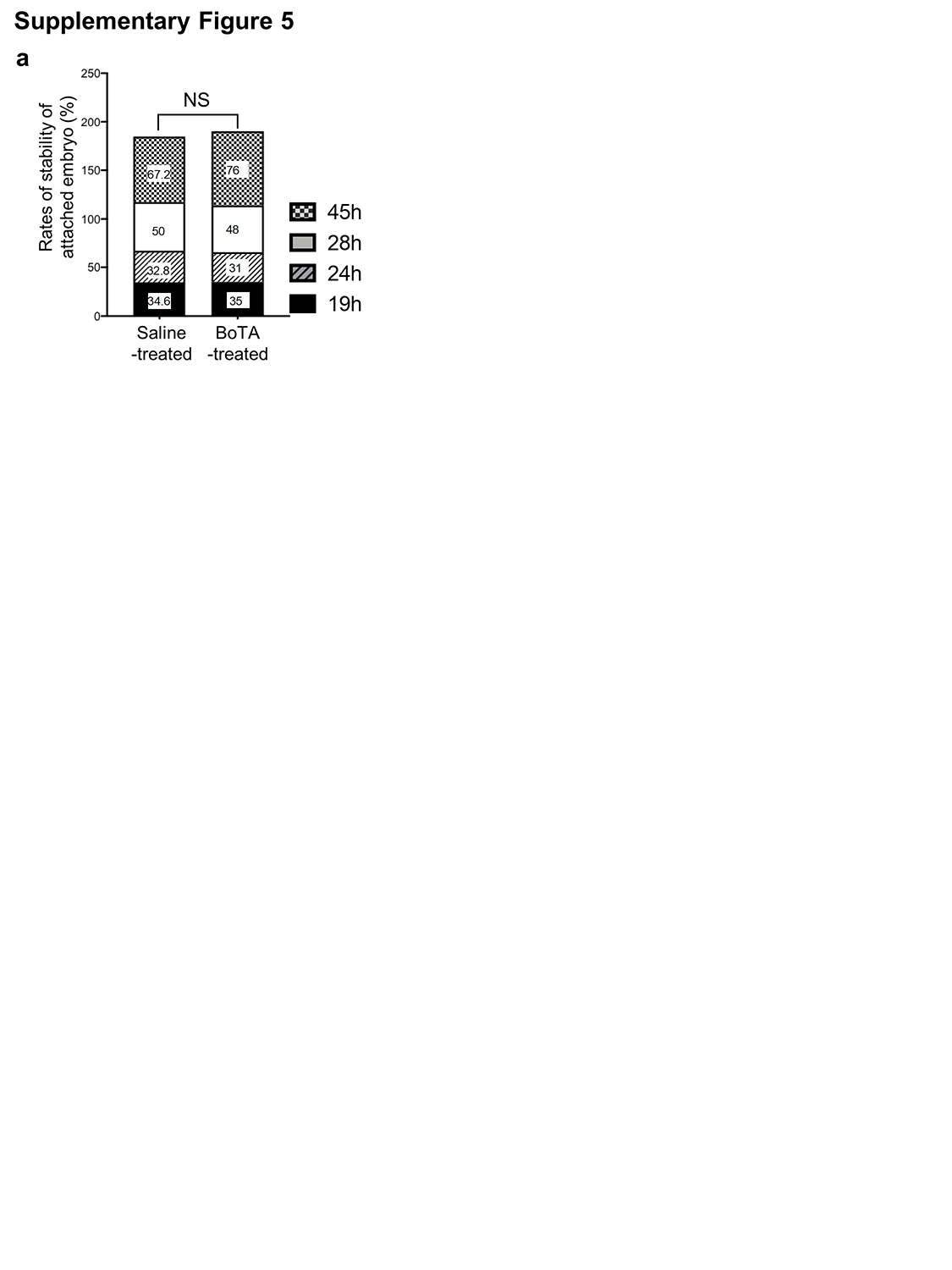

Supplement: Supplementary file 9 — Embryo attachment in non-primed BoTA-treated Ishikawa cells, (a) Rates of stability of attached mouse embryos onto saline- or non-primed BoTA-treated Ishikawa cells at 19h, 24h, 28h, or 45h of co-culture. (PNG 4948 kb). [file 43032_2021_496_Fig11_ESM.png]

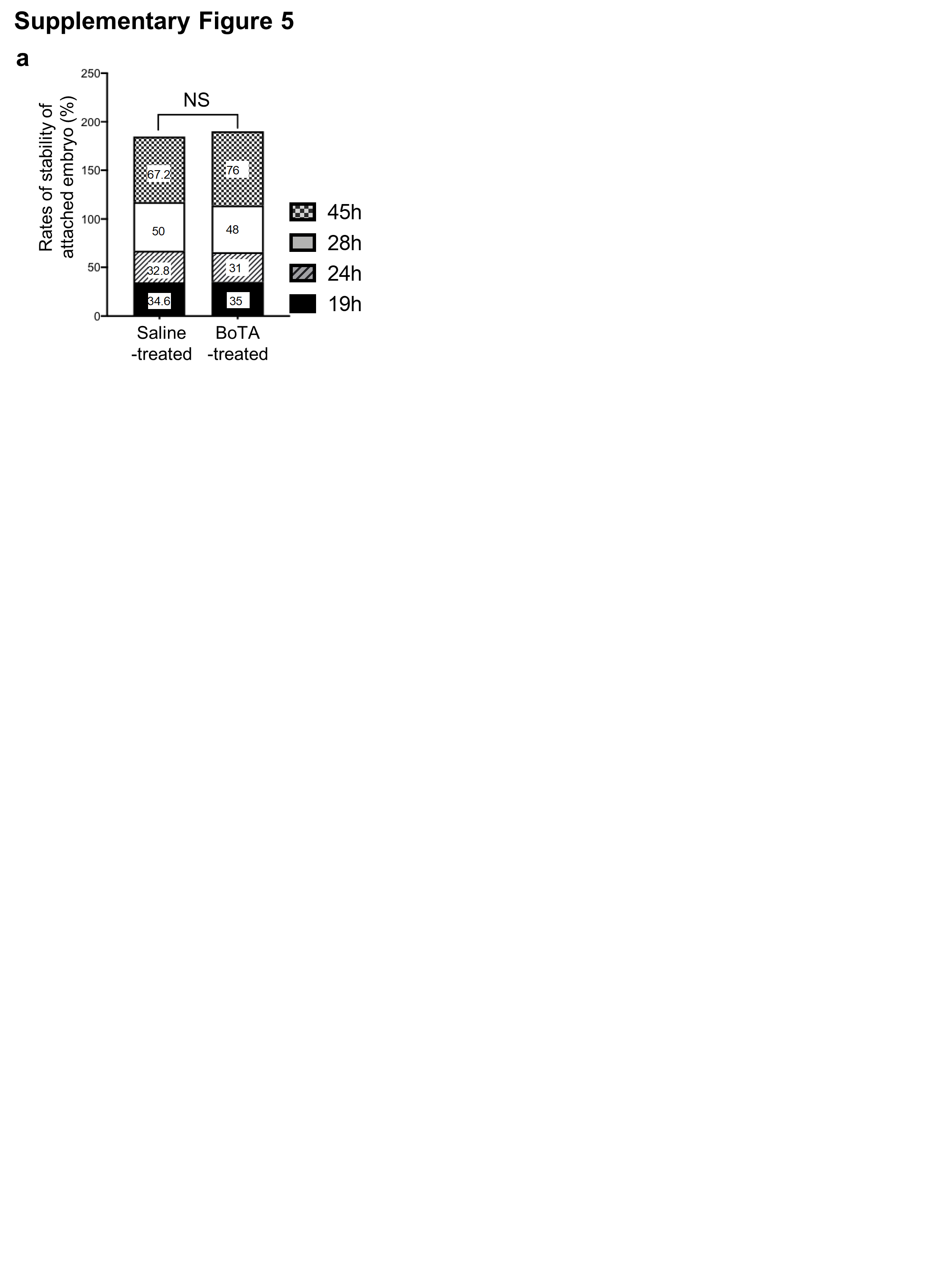

Supplement: Supplementary file 10 — High resolution image (TIF 537 kb). [file 43032_2021_496_MOESM5_ESM.tif]
